# Comparative chloroplast genome analysis of four *Polygonatum* species insights into DNA barcoding, evolution, and phylogeny

**DOI:** 10.1038/s41598-023-43638-1

**Published:** 2023-10-01

**Authors:** Meixiu Yan, Shujie Dong, Qiuyi Gong, Qin Xu, Yuqing Ge

**Affiliations:** 1 The First Affiliated Hospital of Zhejiang Chinese Medical University, 54 Youdian Road, Hangzhou, Zhejiang Province People’s Republic of China; 2 School of Pharmaceutical Sciences, Zhejiang Chinese Medical University, Hangzhou, People’s Republic of China

**Keywords:** Genetics, Plant sciences

## Abstract

The *Polygonatum* genus represents a perennial herb with the Liliaceae family, boasting substantial economic and medicinal significance. The majority of *Polygonatum* plants exhibit notable similarity while lacking distinctive identifying characteristics, thus resulting in the proliferation of adulterated medicinal materials within the market. Within this study, we conducted an in-depth analysis of the complete chloroplast (cp) genomes of four *Polygonatum* plants and compared them with four closely akin species. The primary objectives were to unveil structural variations, species divergence, and the phylogenetic interrelations among taxa. The cp genomes of the four *Polygonatum* species were typified by a conventional quadripartite structure, incorporating a large single copy region (LSC), a small single copy region (SSC), and a pair of inverted repeat regions. In total, we annotated a range of 131 to 133 genes, encompassing 84 to 86 protein-coding genes, 38 transfer RNA (tRNA) genes, 8 ribosomal RNA (rRNA) genes, and 0 to 2 pseudogenes (*ycf1*, *infA*). Our comparative analyses unequivocally revealed a remarkable consistency in gene order and GC content within the *Polygonatum* genus. Furthermore, we predicted a potential 59 to 64 RNA editing sites distributed across 22 protein-coding genes, with the *ndhB* gene exhibiting the most prominent propensity for RNA editing sites, boasting a tally of 15 sites. Notably, six regions of substantial potential variability were ascertained, characterized by elevated Pi values. Noteworthy, molecular markers for species identification, population genetic scrutiny, and phylogenetic investigations within the genus were identified in the form of the *psaJ-rpl33* and *trnS* + *trnT-psaD* barcodes. The resultant phylogenetic tree unequivocally depicted the formation of a monophyletic clade comprising species within the evolutionary framework of Liliaceae, demonstrating closer evolutionary affinities with *Maianthemum*, *Dracaeneae*, and *Asparageae*. This comprehensive compendium of findings collectively contributes to the advancement of molecular species identification, elucidation of phylogenetic interrelationships, and the establishment of DNA barcodes tailored to the *Polygonatum* species.

## Introduction

The *Polygonatum* genus, a perennial herbaceous entity, finds its taxonomic affiliation within the Polygonateae tribe of the Liliaceae family. It holds considerable promise within the realms of the food industry and medicinal research. A rich inventory of 71 species, coupled with several variations, populates this genus, with China accounting for the identification of 39 distinct taxa. These plants favour environments characterized by moisture and shade, thriving in regions endowed with robust, fertile soil. Their habitat spans across the temperate Northern Hemisphere ^1^. Notably, it's significance extends to the Chinese domain, as the source plants for two constituents of Chinese Materia Medica, namely “Huangjing” and “Yuzu”, trace their origins to this genus. The rhizomatous parts of these plants serve as fundamental components of traditional medicinal and functional food preparations^2^. The Chinese Pharmacopoeia of 2020, designates *P. sibiricum*, *P. cyrtonema*, and *P. kingianum*, as the source plants for “Huangjing”, while “Yuzhu” corresponds to *P. odoratum*. A multitude of the *Polygonatum* species contain diverse bioactive compounds, encompassing polysaccharides, steroidal saponins, lectin polysaccharides, and a range of amino acids beneficial, all offering potential benefits to human health^3^. Contemporary pharmacological explorations have further demonstrated the global therapeutic efficacy of agents derived from the *Polygonatum* species against various ailments, including but not limited to fatty liver conditions, Alzheimer’s disease^4^, obesity^5^, and multiple forms of cancer^6–8^. The present, scenario manifests instability in foliar epidermal traits and phyllotactic characteristics of the *Polygonatum* species. This instability impedes swift and precise classification based solely on morphological attributes. The intricate web of variations and interspecies hybridization within the *Polygonatum* genus adds complexity to the process of taxonomy construction. Therein lies the intrigue of gathering comprehensive molecular insights into *Polygonatum* plants, envisaging their contribution to a robust strategy for medicinal material identification and enhancing our comprehension of interspecies relationships within the *Polygonatum* genus. Within our local sphere of medicinal materials commerce, *P. filipes*, *P. cyrtonema*, *P. zanlanscianense*, and *P. odoratum* emerge as the most prevalent species. Notably, their morphological characteristics exhibit minimal differentiation, posing a challenge to identification endeavours. Consequently, an imperative rests upon the determination of complete cp genomes and complete cp genomes and the ensuing comprehensive analysis of cp structures across the quartet of the *Polygonatum* species. Such an endeavour bears substantial significance, elucidating the evolutionary and phylogenetic links while simultaneously unveiling potential molecular markers that could facilitate species identification.

Various species and plants encompassed within the genus *Polygonatum* have long been employed in the realms of traditional medicine and functional nutrition^1^. However, due to their marked similarity and lack of distinctive identifying attributes, discerning *Polygonati rhizoma* and *Rhizoma Polygonati Odorati* from potential root-based adulterants within the *Polygonatum* genus posed considerable challenges. The phenotypic traits exhibited by *Polygonatum* rhizomes could be significantly influenced by their growth dynamics and traditional processing methods, leading to potential misidentification of Huangjing and Yuzhu, both possessing analogous rhizome characteristics. A noteworthy illustration involves the dried rhizomes of *P. odoratum, P. prattii*, and *Disporopsis fuscopicta*, which were often interchangeably misconstrued, thereby giving rise to spurious products that stood in as substitutes for *Polygonati Odorati Rhizoma* within the market^9^. *P. filipes* or *P. zanlanscianense* has often mixed with *Polygonati Rhizoma* and served as a potential adulterant in the medicinal material market^10^, leading to significant challenges in classic morphological identification^11^. The current sources of *Polygonatum* are mostly artificially cultivated, and it is necessary to select authentic varieties of *Polygonatum* for domestication and planting. Different species of *Polygonatum* have different components and potencies, and a method is needed to distinguish the authenticity of *Polygonatum*. From the above analysis, the emergence of substitutes and fake products of *Polygonati Rhizoma* and *Rhizoma Polygonati Odorati* have brought potential health and safety risks, and an effective identification strategy is needed to ensure the quality of *Polygonatum* herbs.

Within plant cells and eukaryotic algae, the chloroplast (cp) assumes the role of a principal plasmid and semi-autonomous organelle. Characterized by a profoundly, conserved structure, it comprises a large single copy (LSC) region, a small single-copy (SSC) region, and two inverted repeat (IR) regions , namely IRa and IRb^12^. In comparison to the nuclear genome, the cp genome manifests a lower mutation rate and a more stable mode of inheritance, rendering it fitting medium for the study of plant phylogenetics and species discernment^13^. The repository of complete cp genomes has substantially enriched our comprehension of plant biology and evolution, amplifying the molecular tools available for species identification and facilitating the cultivation of valuable medicinal plants^14^. Scrutiny of chloroplast coding regions through molecular phylogenetic analyses has confirmed the monophyletic nature of *Polygonatum* and *Heteropolygonatum*, thereby reinforcing their classification as distinct genera^15^. Furthermore, in-depth comparative analysis and phylogenetic examination of 26 species' cp genomes within the realm of *Polygonatum* and Tribal *Polygonatum* have yielded invaluable insights into the interrelationships among the genera encompassed by Trib. Polygonateae^16^. Beyond their utility in phylogenetic reconstruction and evolutionary inquiry, cp genomes possess the potential to emerge as potent assets in the authentication of medicinal plants and their closely allied or adulterated counterparts. Elucidated through a maximum likelihood tree rooted in comprehensive cp sequences, *Dendrobium officinale* and its closely affiliated species demonstrated optimal discrimination, unveiling an efficacious molecular tactic for validating *Dendrobium* taxa^17^. Our antecedent investigations have underscored that DNA barcodes specifically derived from the complete cp genome of *Stephania tetrandra* could be harnessed as molecular markers for species identification and even a prospective strategy for geographical within *Stephania* species^18^. Therefore, it is essential to determine the complete cp genomes of *Polygonatum* species and undertaking a comparative evaluation bears the potential to emerge as a pivotal tool for the taxonomic appraisal of *Polygonatum* plants, further contributing to the harmonization of the market of *Polygonatum*.

*P. filipes*, *P. cyrtonema*, *P. zanlanscianense* and *P. odoratum* stand as four important species within the genus *Polygonatum*, enjoying widespread cultivation across several provinces in China, where they are utilised both as medicinal resources and culinary fare. In the present investigation, we embarked on a comprehensive analysis involving the sequencing and assembly of the complete cp genomes of these four significant *Polygonatum* species. Employing a next-generation sequencing platform, we delved into the intricate phylogenetic connections and taxonomic positioning of *Polygonatum*, leveraging the entirety of the cp genomes. This study endeavoured to achieve several goals: (i) a comparative exploration of the *Polygonatum* cp genomes, yielding insights into fundamental genome architecture, codon usage bias, repetitive structural attributes, and sites subject to RNA editing; (ii) the identification of pivotal regions of interest, thus setting the stage for the formulation of potential DNA markers, primed to efficaciously differentiate various plants; (iii) the establishment of a comprehensive phylogenetic framework delineating the relationships amongst species, thereby facilitating inferences about the taxonomic stature of *Polygonatum* within the subfamily Liliaceae, derived from the wealth of data furnished by complete cp gene sequences. The outcomes of this inquiry disseminate substantial intelligence concerning *Polygonatum* plants, furnishing crucial information for investigations into taxonomical classification, genetic diversity across distinct populations, and the discrimination of closely related species within the genus *Polygonatum*. Ultimately, the exhaustive analysis of cp genomes bears the potential to standardize the cultivation and propagation of foundational raw materials from *Polygonatum*.

### Ethics approval and consent to participate

All the plant materials in this study had obtained permission from the Xincheng Agriculture, Forestry and Animal Husbandry Professional Cooperative of Sui Chang County and Pukang Chinese Herbal Medicine Planting Base of Bozhou City, Anhui Province. The plant material collection and experimental research were conducted in accordance with the Plant Protection and Regulation of Zhejiang Chinese Medical University.

## Results

### Chloroplast genome features of four *Polygonatum* species

**Figure 1:**
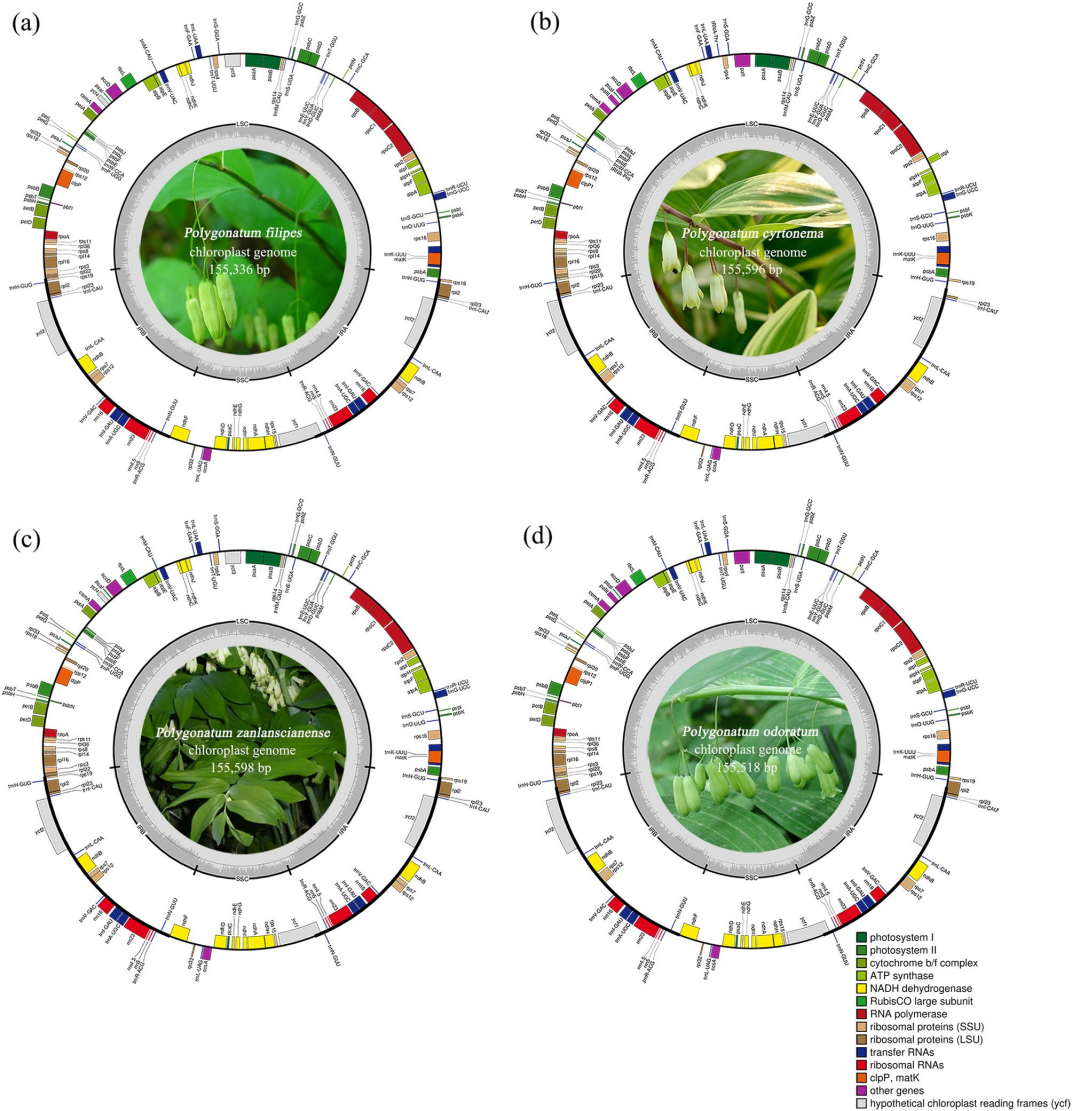
Chloroplast maps of four *Polygonatum* species (**a**) *P. filipes*, (**b**) *P. cyrtonem*a, (**c**) *P. zanlanscianense*, (**d**) *P. odoratum*. Genes shown outside the outer circle are transcribed clockwise, and those inside are transcribed counterclockwise. The darker gray of the inner circle indicates the GC content and the lighter gray indicates the AT content. Genes with different colors encode different functions. The figure shows a large single copy (LSC) region, the inverted repeat (IR) region and a small single copy (SSC) region.

In this phase of our study, we pursued the sequencing of four *Polygonatum* species namely *P. filipes*, *P. cyrtonema*, *P. zanlanscianense* and *P. odoratum*, using the Illumina HiSeq 2500 sequencing platform. This endeavour yielded a corpus of Illumina reads ranging from 15,915,998 to 32,147,396, coupled with an overall sequence length of 3,329,565,273 to 4,600,981,992 bases. Following a meticulous process of read trimming, selection, and assembly, the cp genomes of these four *Polygonatum* species were successfully constructed. Their lengths spanned from 155,336 to 155,598 base pairs (Fig. 1), mirroring the reported cp genome sizes of other plants within *Polygonatum*, such as *P. involucratum* (OL405015: 155,372 bp) and *P. zanlanscianense Pamp.* (MW800891: 155,609 bp)^16,19^. Reminiscent of the characteristic architecture pervasive within angiosperms^20,21^, the four *Polygonatum* species adhered to the conventional four-part configuration, consisting a large single copy region (LSC: 84,282–87,210 bp), a small single-copy region (SSC: 18,292–18,454 bp), and a pair of inverted repeat regions (IRs: 25,008–26,372 bp) that demarcated the LSC and SSC domains. Our repository of the four *Polygonatum* cp genome sequences has been duly deposited in the GenBank under the accession numbers MZ571521, MZ579646, MZ568930, and MZ666387. As depicted in Table 1, the cp genomes of these four *Polygonatum* species collectively encoded a range of 131–133 genes, encompassing 84–86 protein-coding genes, 38 transfer RNA (tRNA) genes, 8 ribosomal RNA (rRNA) genes, and 0–2 pseudogenes. Despite rigorous efforts to mitigate annotation errors by cross-referencing with existing cp genome databases, variations in the total gene count and the number of protein-coding genes within the same genus persisted. However, in consonance with the gene counts of *Polygonatum* species as documented by the NCBI, *P. sibiricum* (NC_029485), and *P. verticillatum* (NC_028523) recorded totals of 136 genes, while *P. kingianum* (NC_047406) featured 130 genes. Additionally, *P. cyrtonema* (NC_028429) comprised 83 coding genes, whereas both *P. macropodum* (MZ150854) and *P. acuminatifolium* (MZ150867) contained a count of 87 coding genes. The intrageneric variability observed in the aforementioned gene counts may be attributed to the considerable diversity in species, structural divergence, and genetic dynamics inheren within the genus *Polygonatum*. Intriguingly, within the comprehensive ensemble of the *Polygonatum* cp genome genes, 24 exhibited the presence of introns. Among these, with 11 protein-coding genes (*petB*, *petD*, *atpF*, *ndhA*, *ndhB (*× *2)*, *rpoC1*, *rps16*, *rpl2 (*× *2)*, *rpl16*) and 8 tRNA genes (*trnK-UUU*, *trnG-UCC*, *trnL-UAA*, *trnV-UAC*, *trnI-GAU (*× *2)* and *trnA-UGC (*× *2)*) displayed a singular intron, while 5 protein-coding genes (*rps12 (*× *2)*, *pafI*, *clpP*, *ycf3*) featured two introns each. Notably, the gene *pafI*, present in *P. cyrtonema* and *P. odoratum* exhibited a relative absence in other *Polygonatum* plants^22^. Conversely, *ycf3* manifested within *P. filipes* and *P. zanlanscianense*, a characteristic more prevalent across most of the *Polygonatum* genus^23^. Subtle disparities surfaced in the repertoire of protein-coding genes and pseudogenes across the *Polygonatum* cp genomes. For instance, while the cp genomes of *P. filipes* and *P. zanlanscianense* retained the *ycf3* and *ycf4* genes, *P. cyrtonema* and* P. odoratum* exhibited the loss of these two genes. Similarly, the genes *pafI* and *pafII* genes were exclusive to *P. cyrtonema* and *P. odoratum* (Table 1)*.* Moreover, the *infA* gene, responsible for encoding the translation initiation factor, was identified as a pseudogene, albeit absent from the *P. odoratum* cp genome. Remarkably, *P. filipes* exhibited two pseudogenes, namely *ycf1* and *infA*, while *P. cyrtonema* and *P. zanlanscianense* contained only an intact *infA* gene within their plastid structures.

**Table 1:** List of genes annotated in the chloroplast genomes of four *Polygonatum* species.

No	Group of gene	Gene names	Amount
1	Photosystem I	*psaA, psaB, psaC, psaI, psaJ*	5
2	Photosystem II	*psbA, psbB, psbC, psbD, psbE,psbF, psbH, psbI, psbJ, psbK,psbL, psbM, psbT, psbZ*	14
3	Cytochrome b/f complex	petA, petB*, petD*, petG, petL, petN	6
4	ATP synthase	atpA, atpB, atpE, atpF*, atpH, atpI	6
5	NADH dehydrogense	ndhA*, ndhB*(× *2), ndhC, ndhD, ndhE, ndhF, ndhG, ndhH, ndhI, ndhJ, ndhK*	12
6	RubisCO large subunit	*rbcL*	1
7	RNA polymerase	rpoA, rpoB, rpoC*1, rpoC2	4
8	Ribosomal proteins (SSU)	*rps2,rps3,rps4,rps7(*× 2),rps8,rps11,rps12**(× 2),rps14,rps15, rps16*, rps18, rps19(× *2)*	15
9	Ribosomal proteins (LSU)	rpl2*(× 2), rpl14, rpl16*, rpl20, rpl22, rpl23(× *2),rpl32, rpl33, rpl36*	11
10	Other genes	accD,ccsA,cemA,clpP**, matK, pafI**^,pafII^, pbf1	6–8
11	Proteins of unknown function	*ycf1, ycf2(*× 2), ycf3**#, ycf4#	3–5
12	Transfer RNAs	*38 tRNAs* (8 contain an intron,8 in the IRs)	38
13	Ribosomal RNAs	*rrn4.5(*× *2), rrn5(*× *2), rrn16(*× *2), rrn23(*× *2)*	8
14	pseudo genes	*ycf1, infA*	2

One or two asterisks after a gene indicate that the gene contains one or two introns, respectively.

^#^*ycf3* gene only appears in *Polygonatum filipes*, *ycf4* gene appears in *Polygonatum filipes* and *Polygonatum zanlanscianense*.

^ *pafI*, *pafII* appear in *Polygonatum cyrtonema* and *Polygonatum odoratum.*

The cp complete genomes of the four *Polygonatum* species exhibited remarkable similarities concerning gene composition, overall length, and GC content. Specifically, the global GC content measured at 37.68% for *P. filipes*, 37.69% for *P. cyrtonema*, 37.66% for *P. zanlanscianense*, and 37.70% for *P. odoratum*, revealing a near-identical pattern across these species (Table 2). This uniformity was also evident when juxtaposed with the GC content of other members within the Liliaceae family, such as *Maianthemum dilatatum* and *Dracaena draco*, where GC contents were observed at 37.59% and 37.60%, respectively. However, it is noteworthy that the distribution of GC content diverged across distinct regions within the cp genomes. Notably, the IR regions commanded the highest GC content within the four cp genomes. This phenomenon could be attributed to the high GC content intrinsic to rRNA sequences present within the IR regions. In the spectrum of protein-coding genes, deviations were apparent in the initiation codons of the *rps19* and *rpl2* genes, which varied from the conventional start codon ATG. Specifically, the *rps19* and *rpl2* genes in the four species commenced with GTG and ATA, respectively. An intriguing revelation pertained to the presence of *pafI* and *pafII* genes within the cp genomes of *P. cyrtonema* and *P. odoratum*, juxtaposed with the absence of *ycf3* and *ycf4* genes. This marked distinction underscored the divergence between *P. cyrtonema* and *P. odoratum* from the other two *Polygonatum* species. Cumulatively, these findings elucidated nuanced differentiations within the cp genomes of diverse *Polygonatum* species, while simultaneously highlighting their shared foundations encompassing fundamental structural elements, genome dimensions, and total GC content.

**Table 2:** Statistics on the basic feature of the chloroplast genomes of *Polygonatum* species.

Characteristics	*P. filipes*	*P. cyrtonema*	*P. zanlanscianense*	*P. odoratum*	*P. sibiricum*	*P. humile*	*P. kingianum*	*P. cirrhifolium*
Accession number	MZ571521	MZ579646	MZ568930	MZ666387	NC_029485	MN218691	MW566468	NC_053687
Total length(bp)	155,336	155,596	155,598	155,518	152,960	155,313	155,795	155,583
LSC length(bp)	84,282	87,020	84,428	87,210	81,539	84,239	84,613	84,412
SSC length(bp)	18,454	18,404	18,426	18,292	18,519	18,470	18,530	18,427
IR length(bp)	26,300	25,086	26,372	25,008	26,451	26,302	26,326	26,372
Total Number of Genes	133	131	133	131	136	131	134	131
Coding Genes	85	84	86	85	84	85	84	85
rRNA Genes	8	8	8	8	8	8	8	8
tRNA Genes	38	38	38	38	38	38	38	37
Pseudogenes	2( *ycf1, infA*)	1(*infA*)	1(*infA*)	0	6(infA,ycf1,ycf15*,ycf68*)	0	4(infA,ycf1,ycf15*)	1( *ycf1*)
Total GC content (%)	37.68	37.69	37.66	37.70	37.95	37.69	37.66	37.66
LSC GC content (%)	35.71	35.85	35.70	35.87	35.96	35.73	35.70	35.71
SSC GC content (%)	31.56	31.65	31.55	31.69	32.10	31.53	31.59	31.55
IR GC content (%)	42.98	43.10	42.92	43.08	43.05	42.99	42.96	42.92

*Indicates a duplicated gene.

LSC: large single copy, SSC: small single copy, IR: inverted repeat, GC: guanine and cytosine.

### Codon biased usage analysis

**Figure 2:**
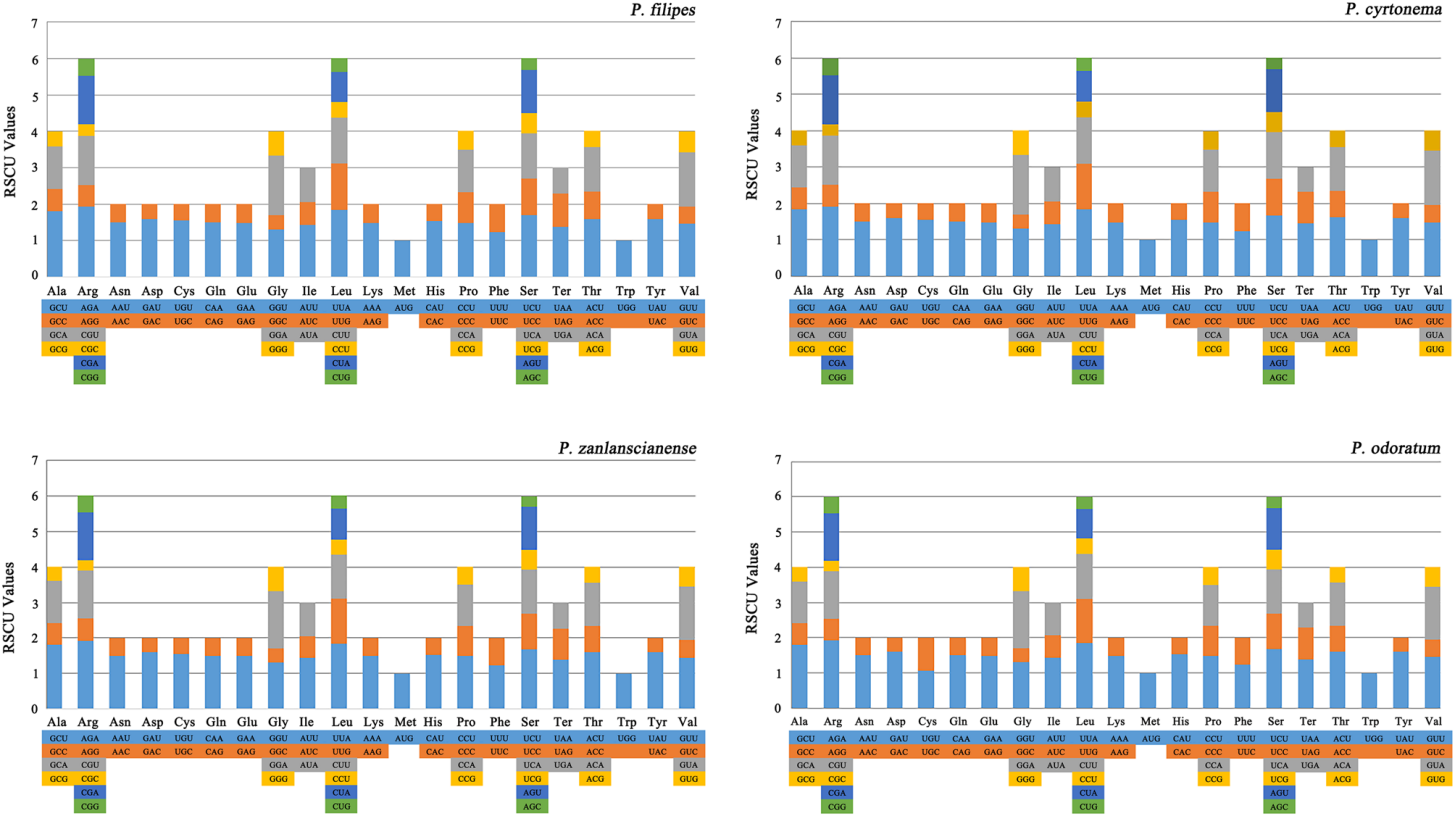
The codon content and RSCU value of 20 amino acids and stop codons in all protein-coding genes in the chloroplast genomes of four *Polygonatum* species. The color of the histogram is consistent with the color of codons.

Further delving into the intricacies of codon usage, we proceeded to ascertain the frequency of codon usage and the associated relative synonymous codon usage (RSCU) values within the four *Polygonatum* cp genomes. The cumulative coding sequence lengths within spanned from 77,940 bp (*P. cyrtonema*) to 79,653 bp (*P. zanlanscianense*), with *P. odoratum* and *P. filipes* encompassing 78,679 bp and 78,687 bp, respectively. Within these sequences, the number of protein-coding genes in the four *Polygonatum* species, ranged from 84 to 86, encompassing codon counts that varied from 25,980 (in *P. cyrtonema*) to 26,551 (in *P. zanlanscianense*). For comparative analysis, we also selected four *Polygonatum* plants with the same species names from GenBank, namely *P. filipes* (MZ150843), *P. cyrtonema* (MZ150839), *P. zanlanscianense* (MW800891), and *P. odoratum* (MZ150859), for an in-depth codon study. The coding sequences of these plants spanned from 78,817 bp to 79,233 bp, housing 86 protein-coding genes with codon tallies ranging from 26,226 (*P. zanlanscianense*) to 26,424 (*P. cyrtonema*), aligning closely with our sequencing findings. Codon analysis spotlighted the AUU codon encoding isoleucine (Ile) as the most prominently used, tallying at 1096 occurrences *P. zanlanscianense*. On the contrary, the UGC codon encoding cysteine (Cys) stood as the least employed, registering a count of 68 in *P. odoratum* (Table 3). The RSCU, a metric that gauges the ratio of codon usage frequency to theoretical frequency^24^, displayed a trend of preference for all amino acids except for methionine (AUG) and tryptophan (UGG), which were represented by a solitary codon (RSCU = 1) (Fig. 2, Table 3). Broadly, when RSCU > 1, it signified a preference for the codon in question^25^. The spectrum encompassed 31 preferred (RSCU > 1) and 31 non-preferred (RSCU < 1) synonymous codons were determined. The AGA codon held the highest RSCU value of 1.93, whereas the CGC codon demonstrated the lowest value of 0.30. Intriguingly, within GenBank, *P. zanlanscianense* (MW800891) emerged as a distinctive outlier with the highest RSCU value of 2.11, differing markedly from other *Polygonatum* plants. Another notable departure was observed with *P. filipes* (MZ150843), where AGC, rather than CGC codon, exhibited the lowest RSCU value. A fascinating trend emerged among the 31 codons with RSCU > 1: 28 of these codons terminated with A/U, in stark contrast to a majority of C/G-ending codons with RSCU values falling below 1 (Table 3). This observation indicates a pronounced preference for the third codon A/U within the coding genes of, surpassing the preference for C/G codons. Additionally, the arginine-encoding AGA codon featured an RSCU value of 1.93, closely shadowed by its counterpart in *P. cyrtonema*, which registered at 1.91. Collectively, this analysis underscored a marked level of conservatism in codon usage preferences observed within the cp genomes of the four *Polygonatum* species.

**Table 3:** Codon usage and codon-anticodon recognition patterns of four *Polygonatum* plants.

Codon	tRNA	Numbers and RSCU			
		*P. filipes*	*P. cyrtonema*	*P. zanlanscianense*	*P.odoratum*
UUU(F)		915/1.23	919/1.23	934/1.23	918/1.23
UUC(F)	*trnF-GAA*	576/0.77	574/0.77	585/0.77	575/0.77
UUA(L)		827/1.85	816/1.84	842/1.85	827/1.85
UUG(L)	*trnL-CAA*	561/1.26	562/1.26	568/1.25	563/1.26
CUU(L)		565/1.27	563/1.27	569/1.25	568/1.27
CUC(L)		189/0.42	191/0.43	197/0.43	193/0.43
CUA(L)		376/0.84	372/0.84	385/0.85	375/0.84
CUG(L)		161/0.36	162/0.36	166/0.37	162/0.36
AUU(I)		1086/1.43	1081/1.44	1096/1.44	1085/1.44
AUC(I)	*trnI-GAU*	469/0.62	463/0.62	470/0.62	467/0.62
AUA(I)		719/0.95	706/0.94	723/0.95	716/0.95
AUG(M)	*trnM-CAU*	620/1.00	619/1.00	634/1.00	620/1.00
GUU(V)		516/1.46	514/1.47	522/1.45	519/1.46
GUC(V)		171/0.48	170/0.48	178/0.49	173/0.49
GUA(V)		527/1.49	525/1.50	540/1.50	531/1.50
GUG(V)		198/0.56	194/0.55	201/0.56	196/0.55
UCU(S)		578/1.69	569/1.67	584/1.67	575/1.67
UCC(S)	*trnS-GGA*	347/1.01	350/1.02	353/1.01	352/1.02
UCA(S)	*trnS-UGA*	427/1.24	429/1.26	435/1.25	428/1.25
UCG(S)		191/0.56	190/0.56	196/0.56	192/0.56
CCU(P)		404/1.49	398/1.48	408/1.49	403/1.48
CCC(P)		229/0.84	229/0.85	231/0.84	233/0.86
CCA(P)	*trnP-UGG*	317/1.17	310/1.15	319/1.16	315/1.16
CCG(P)		138/0.51	137/0.51	140/0.51	138/0.51
ACU(T)		535/1.60	532/1.62	539/1.60	537/1.61
ACC(T)	*trnT-GGU*	246/0.74	237/0.72	248/0.74	243/0.73
ACA(T)	*trnT-UGU*	406/1.22	399/1.22	410/1.22	406/1.22
ACG(T)		149/0.45	145/0.44	151/0.45	148/0.44
GCU(A)		633/1.80	636/1.84	638/1.81	634/1.81
GCC(A)		217/0.62	206/0.59	217/0.62	214/0.61
GCA(A)		413/1.17	403/1.16	414/1.18	410/1.17
GCG(A)		144/0.41	141/0.41	139/0.39	142/0.41
UAU(Y)		756/1.59	749/1.59	768/1.59	753/1.59
UAC(Y)	*trnY-GUA*	196/0.41	191/0.41	201/0.41	196/0.41
UAA(*)		39/1.38	42/1.45	40/1.40	39/1.38
UAG(*)		26/0.92	25/0.86	25/0.87	26/0.92
CAU(H)		514/1.53	507/1.54	519/1.53	515/1.53
CAC(H)	*trnH-GUG*	156/0.47	153/0.46	159/0.47	157/0.47
CAA(Q)	*trnQ-UUG*	701/1.51	690/1.50	698/1.50	695/1.51
CAG(Q)		229/0.49	227/0.50	230/0.50	228/0.49
AAU(N)		949//1.51	938/1.51	961/1.51	947/1.51
AAC(N)		307/0.49	302/0.49	313/0.49	307/0.49
AAA(K)		1010/1.49	995/1.48	1032/1.49	1010/1.48
AAG(K)		347/0.51	347/0.52	351/0.51	351/0.52
GAU(D)		862/1.60	856/1.60	866/1.60	861/1.59
GAC(D)	*trnD-GUC*	217/0.40	215/0.40	219/0.40	221/0.41
GAA(E)	*trnE-UUC*	1011/1.49	1007/1.48	1035/1.49	1014/1.48
GAG(E)		349/0.51	352/0.52	350/0.51	354/0.52
UGU(C)	*trnC-GCA*	239/1.55	237/1.55	240/1.54	235/1.55
UGC(C)		69/0.45	69/0.45	71/0.46	68/0.45
UGA(*)		20/0.71	20/0.69	21/0.73	20/0.71
UGG(W)	*trnW-CCA*	461/1.00	461/1.00	464/1.00	461/1.00
CGU(R)	*trnR-ACG*	366/1.35	361/1.35	369/1.35	366/1.35
CGC(R)		85/0.31	81/0.30	84/0.31	83/0.31
CGA(R)		364/1.34	361/1.35	363/1.33	361/1.33
CGG(R)		125/0.46	126/0.47	129/0.47	127/0.47
AGU(S)		409/1.19	405/1.19	416/1.19	408/1.19
AGC(S)	*trnS-GCU*	106/0.31	107/0.31	108/0.31	106/0.31
AGA(R)	*trnR-UCU*	524/1.93	510/1.91	526/1.93	523/1.93
AGG(R)		164/0.60	162/0.61	167/0.61	163/0.60
GGU(G)		579/1.30	570/1.31	587/1.31	582/1.31
GGC(G)	*trnG-GCC*	175/0.39	170/0.39	174/0.39	169/0.38
GGA(G)		727/1.64	714/1.64	732/1.63	726/1.64
GGG(G)		297/0.67	288/0.66	301/0.67	296/0.67

### RNA Editing sites prediction

**Figure 3:**
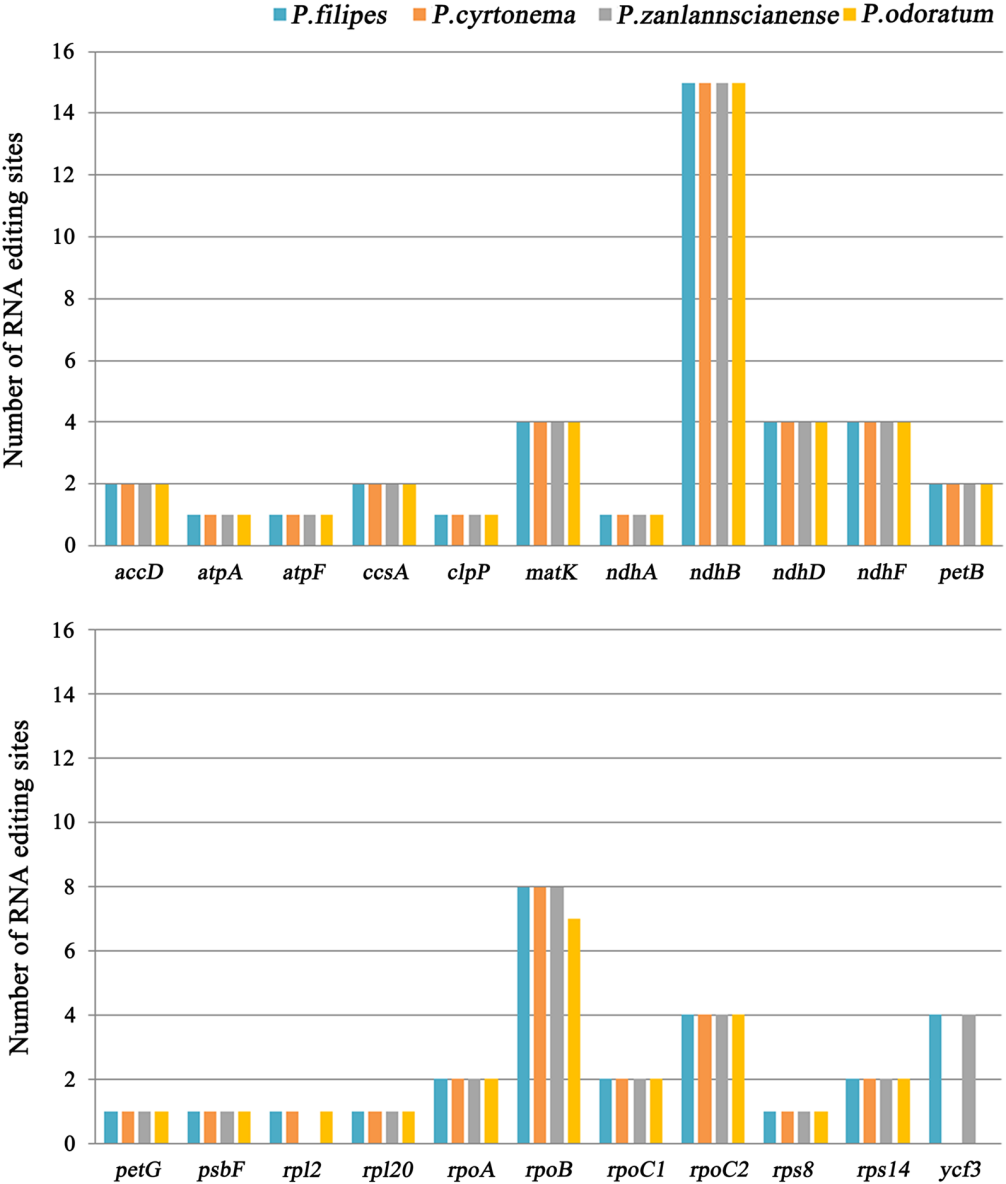
Numbers of RNA editing sites in the chloroplast genomes of four *Polygonatum* species. Different colors represent different species.

In the pursuit of a comprehensive exploration of the *Polygonatum* cp genome, a pivotal facet entails the identification of RNA editing events. This scrutiny led us to predict a total of 59 potential RNA editing sites dispersed across 22 protein-coding genes within the cp genome of *P. odoratum*. Notably, a higher count of sites emerged in *P. filipes* (64 sites), *P. cyrtonema* (60 sites) and *P. zanlanscianense* (63 sites). Amidst the four cp genomes sequenced in our study, the *ndhB* stood out, harboring the most significant tally of potential RNA editing sites (15 editing sites). In close pursuit was the *rpoB* gene (8 sites, with 7 in *P. odoratum*), whereas *atpA*, *atpF*, *clpP*, *ndhA*, *petG*, *psbF*, *rpl2*, *rpl20*, and *rps8* each manifested a solitary editing site (with *rpl2* in *P. zanlanscianense* exhibiting none). Delving further into the specifics, 14 codons were identified to be edited at the first nucleotide position, whilst 50, 46, 49, and 45 codons underwent editing at the second nucleotide position in the cp genomes of *P. filipes*, *P. cyrtonema*, *P. zanlanscianense*, and *P. odoratum*, respectively. The repercussions of these editing sites events encompassed 10 distinct types of amino acid conversions (Table 4). Noteworthy transformations included histidine to tyrosine (H–Y), leucine to phenylalanine (L–F), arginine to tryptophan (R–W), and arginine to cysteine (R–C), stemming from codons edited at the first nucleotide position. Conversely, codons edited at the second nucleotide position resulted in conversions such as serine to leucine (S–L), proline to leucine (P–L), serine to phenylalanine (S–F), threonine to methionine (T–M), alanine to valine (A–V), and threonine to isoleucine (T–I). Of particular note, serine to leucine (S–L) emerged as the most frequent amino acid conversion, accounting for 37.5%, trailed by serine to phenylalanine (S–F, 14.1%), proline to leucine (P–L, 12.5%), and histidine to tyrosine (H–Y, 12.5%) in *P. filipes.* The projection of RNA editing sites within the cp genomes of *P. cyrtonema*, *P. zanlanscianense*, and *P. odoratum* mirrored the findings in *P. filipes* (Fig. 3). A noteworthy observation pertains to the absence of RNA editing sites in the *rpl2* gene of *P. zanlanscianense* and the *ycf3* gene of *P. cyrtonema* and *P. odoratum*, which could exert critical repercussions on the translation and protein activity of these genes (Fig. 3). Given the close connection between RNA editing sites and nucleotide replacements within protein-coding genes, we extended our scrutiny to encompass synonymous substitution (Ks) and nonsynonymous substitution (Ka) in protein-coding genes characterized by abundant RNA editing sites (Table 5). Most genes exhibited Ka/Ks values below 0.5, indicative of discernible purifying selection effects on these protein-coding genes. Particularly striking was the absence of both synonymous and nonsynonymous substitutions in the *rps14*, *petG*, and *psbF* genes across the four *Polygonatum* plants. This phenomenon suggests that these genes might be subject to robust purification pressures and selective forces, rendering them resilient to environmental shifts (Table 5). Of special significance, the *rpl2* gene demonstrated the highest Ka/Ks value of 2.3846 in *P. zanlanscianense* (1.1290 in *P. cyrtonema*, 1.1290 in *P. odoratum*). In a similar vein, the *accD* gene across the four *Polygonatum* species registered Ka/Ks values of 1.0685, 1.4392, 1.0840, and 1.0762, respectively (Table 5), surpassing the value of one, signifying their participation in a diverse selection mode pivotal to the evolutionary pressures shaping. The discernment of RNA editing sites and nucleotide substitutions within protein-coding genes imparts valuable insights into comprehending missense mutations within the cp genomes of *Polygonatum* species.

**Table 4:** Amino acid conversion frequency of protein coding genes in four *Polygonatum* species.

Amino acid conversion	Edited position	Number and percentage			
		*P. filipes*	*P. cyrtonema*	*P. zanlanscianense*	*P. odoratum*
S–L	Second nucleotide	24/37.5%	24/40.0%	24/38.1%	23/39.0%
P–L	Second nucleotide	8/12.5%	7/11.7%	8/12.7%	7/11.9%
H–Y	First nucleotide	8/12.5%	8/13.3%	8/12.7%	8/13.6%
L–F	First nucleotide	3/4.7%	3/5.0%	3/4.8%	3/5.1%
S–F	Second nucleotide	9/14.1%	7/11.7%	9/14.3%	7/11.9%
T–M	Second nucleotide	4/6.3%	3/5.0%	3/4.8%	3/5.1%
A–V	Second nucleotide	1/1.6%	1/1.7%	1/1.6%	1/1.7%
R–W	First nucleotide	2/3.1%	2/3.3%	2/3.2%	2/3.4%
T–I	Second nucleotide	4/6.3%	4/6.7%	4/6.3%	4/6.8%
R–C	First nucleotide	1/1.6%	1/1.7%	1/1.6%	1/1.7%

**Table 5:** The Ka/Ks ratio analysis of protein coding genes with RNA editing sites in four *Polygonatum* species.

Gene	Species	Number of RNA editing sites	Length (bp)	GC content (%)	non-synonymous substitutions (Ka)	synonymous substitutions (Ks)	Ka/Ks
*ndhB*	*P. filipes*	15	3066	37.96	0.0009	0	–
	*P. cyrtonema*	15	3066	37.96	0.0009	0	–
	*P. zanlanscianense*	15	3066	37.90	0.0009	0.0027	0.3333
	*P. odoratum*	15	3066	37.96	0.0009	0	–
*ndhD*	*P. filipes*	4	1521	36.16	0.0043	0.0454	0.0947
	*P. cyrtonema*	4	1521	36.23	0.0052	0.0396	0.1313
	*P. zanlanscianense*	4	1521	36.16	0.0043	0.0396	0.1086
	*P. odoratum*	4	1521	36.16	0.0043	0.0396	0.1086
*rpoB*	*P. filipes*	8	3222	38.73	0.0028	0.0229	0.1222
	*P. cyrtonema*	8	3222	38.76	0.0024	0.0201	0.1194
	*P. zanlanscianense*	8	3222	38.86	0.0028	0.0256	0.1094
	*P. odoratum*	7	3222	38.80	0.0028	0.0201	0.1393
*rpoC2*	*P. filipes*	4	4146	37.22	0.0068	0.0193	0.3523
	*P. cyrtonema*	4	4146	37.31	0.0065	0.0172	0.3779
	*P. zanlanscianense*	4	4146	37.31	0.0087	0.0214	0.4065
	*P. odoratum*	4	4146	37.29	0.0065	0.0172	0.3779
*ndhF*	*P. filipes*	4	2211	33.47	0.0139	0.0448	0.3103
	*P. cyrtonema*	4	2211	33.56	0.0127	0.0469	0.2708
	*P. zanlanscianense*	4	2211	33.38	0.0151	0.0469	0.3220
	*P. odoratum*	4	2211	33.56	0.0127	0.0448	0.2835
*ndhA*	*P. filipes*	1	1098	35.61	0.0037	0.0221	0.1674
	*P. cyrtonema*	1	1098	35.70	0.0024	0.0221	0.1086
	*P. zanlanscianense*	1	1098	35.70	0.0037	0.0258	0.1434
	*P. odoratum*	1	1098	35.70	0.0024	0.0221	0.1086
*matK*	*P. filipes*	4	1563	31.16	0.0091	0.0202	0.4505
	*P. cyrtonema*	4	1563	31.03	0.0091	0.0202	0.4505
	*P. zanlanscianense*	4	1563	31.16	0.0091	0.0144	0.6319
	*P. odoratum*	4	1563	31.09	0.0091	0.0173	0.5260
*atpA*	*P. filipes*	1	1524	40.49	0.0017	0.0274	0.0620
	*P. cyrtonema*	1	1524	40.42	0.0017	0.0246	0.0691
	*P. zanlanscianense*	1	1524	40.29	0.0035	0.0246	0.1423
	*P. odoratum*	1	1524	40.42	0.0017	0.0246	0.1423
*accD*	*P. filipes*	2	1491	34.74	0.6734	0.6302	1.0685
	*P. cyrtonema*	2	1491	34.61	0.3454	0.2400	1.4392
	*P. zanlanscianense*	2	1476	34.96	0.6737	0.6215	1.0840
	*P. odoratum*	2	1491	34.67	0.6777	0.6297	1.0762
*rpoC1*	*P. filipes*	2	2049	39.39	0.0032	0.0147	0.2177
	*P. cyrtonema*	2	2061	39.45	0.0026	0.0126	0.2063
	*P. zanlanscianense*	2	2061	39.50	0.0026	0.0105	0.2476
	*P. odoratum*	2	2058	39.41	0.0026	0.0147	0.1769
*ccsA*	*P. filipes*	2	972	32.72	0.0066	0.0284	0.2324
	*P. cyrtonema*	2	966	32.51	0.3325	0.3768	0.8824
	*P. zanlanscianense*	2	972	32.51	0.0073	0.0455	0.1604
	*P. odoratum*	2	972	32.61	0.0066	0.0333	0.1982
*rpoA*	*P. filipes*	2	1029	37.51	0.0077	0.0130	0.5923
	*P. cyrtonema*	2	1029	37.71	0.0077	0.0130	0.5923
	*P. zanlanscianense*	2	1029	37.61	0.0064	0.0130	0.4923
	*P. odoratum*	2	1029	37.61	0.0064	0.0130	0.4923
*rps8*	*P. filipes*	1	399	35.09	0.0033	0.0319	0.1034
	*P. cyrtonema*	1	399	35.34	0	0.0319	0
	*P. zanlanscianense*	1	399	35.34	0	0.0319	0
	*P. odoratum*	1	399	35.34	0	0.0319	0
*clpP*	*P. filipes*	1	615	41.95	0.0930	0.1594	0.6004
	*P. cyrtonema*	1	615	41.79	0.0021	0.0501	0.0419
	*P. zanlanscianense*	1	615	41.95	0.0021	0.0428	0.0491
	*P. odoratum*	1	615	41.95	0.0021	0.0428	0.0491
*rps14*	*P. filipes*	2	303	41.58	0	0	–
	*P. cyrtonema*	2	303	41.58	0	0	–
	*P. zanlanscianense*	2	303	41.58	0	0	–
	*P. odoratum*	2	303	41.58	0	0	–
*petB*	*P. filipes*	2	648	40.59	0.0242	0.0567	0.4268
	*P. cyrtonema*	2	648	40.43	0.0342	0.0565	0.6053
	*P. zanlanscianense*	2	648	40.43	0	0.0294	0
	*P. odoratum*	2	648	40.43	0.0269	0.0670	0.4015
*petG*	*P. filipes*	1	114	35.09	0	0	–
	*P. cyrtonema*	1	114	35.09	0	0	–
	*P. zanlanscianense*	1	114	35.09	0	0	–
	*P. odoratum*	1	114	35.09	0	0	–
*atpF*	*P. filipes*	1	555	37.84	0.0046	0.0417	0.1103
	*P. cyrtonema*	1	555	37.66	0.0093	0.0504	0.1845
	*P. zanlanscianense*	1	555	37.84	0.0046	0.0417	0.1103
	*P. odoratum*	1	555	37.84	0.0046	0.0417	0.1103
*rpl2*	*P. filipes*	1	1671	44.34	0.0026	0	–
	*P. cyrtonema*	1	837	44.32	0.0035	0.0031	1.1290
	*P. zanlanscianense*	0	1638	44.20	0.0062	0.0026	2.3846
	*P. odoratum*	1	1671	44.34	0.0035	0.0031	1.1290
*rpl20*	*P. filipes*	1	354	37.29	0	0.0119	0
	*P. cyrtonema*	1	354	37.29	0	0.0119	0
	*P. zanlanscianense*	1	354	37.01	0.0037	0.0119	0.3109
	*P. odoratum*	1	354	37.29	0	0.0119	0
*ycf3*	*P. filipes*	4	507	39.64	0	0.0170	0
	*P. cyrtonema*	0	0	0	0	0	–
	*P. zanlanscianense*	4	507	39.64	0.0078	0.0170	0.4588
	*P. odoratum*	0	0	0	0	0	–
*psbF*	*P. filipes*	1	120	42.50	0	0	–
	*P. cyrtonema*	1	120	42.50	0	0	–
	*P. zanlanscianense*	1	120	42.50	0	0	–
	*P. odoratum*	1	120	42.50	0	0	–

### SSRs and long repeats analysis

**Figure 4:**
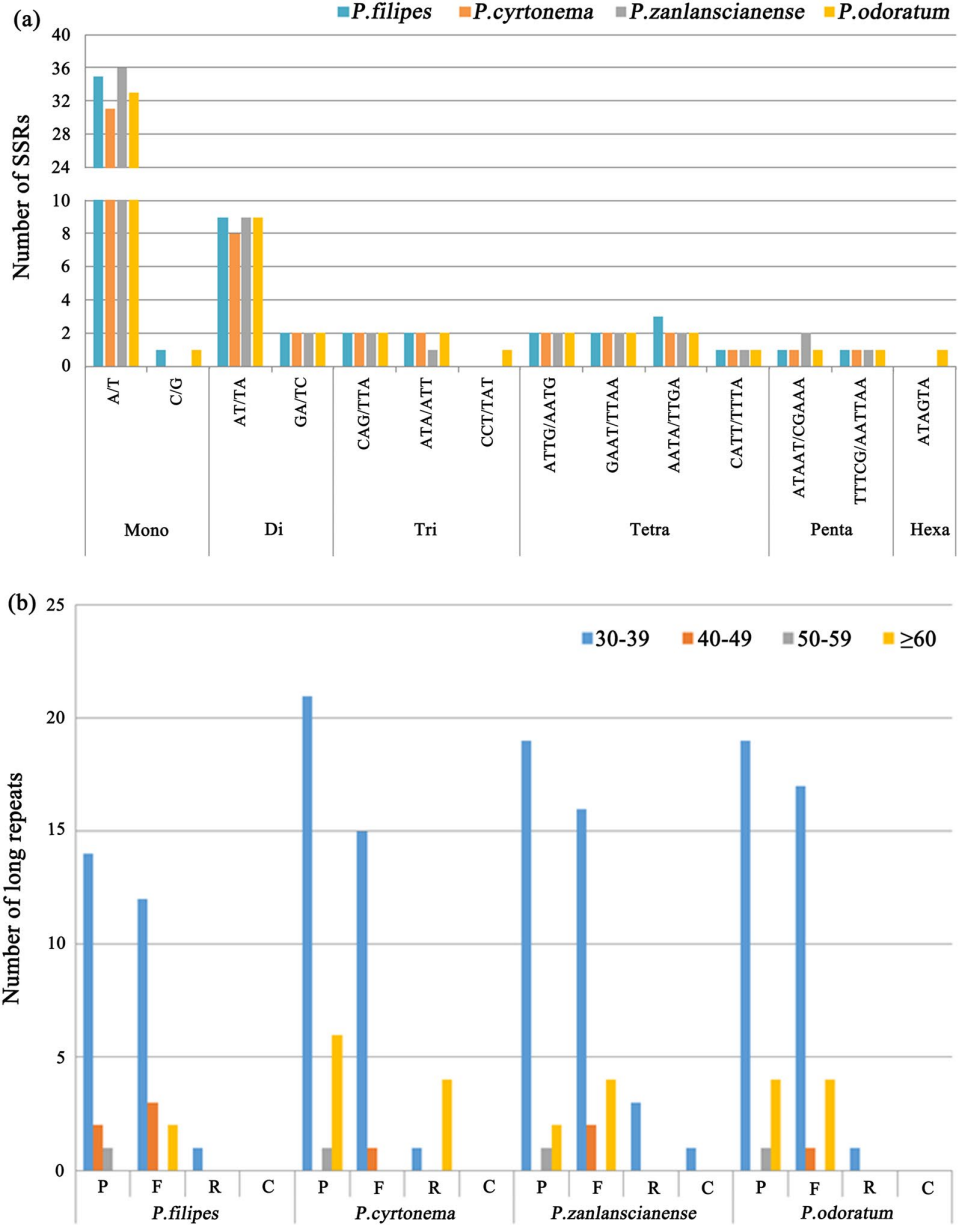
A comparative analysis of repeat sites in four *Polygonatum* plants chloroplast genomes. (**a**) Different types and numbers of SSRs. (**b**) An analysis of long repeats in the number and length. The types of long repeats contained palindromic (P), forward (F), reverse (R) and complement (C).

Within the expansive landscape of the chloroplast genome, Simple sequence repeats (SSRs) exhibit widespread distribution and pronounced polymorphism, offering resilience against environmental impacts. As a potent analytical tool for species identification^26^, this study delved into the spectrum, prevalence, and dispersion of SSRs within the cp genomes of the four *Polygonatum* species. Notably, the count of SSRs totaled 61 in *P. filipes*, 54 in *P. cyrtonema*, and 60 in both *P. zanlanscianense* and *P. odoratum* (Fig. 4a). Among these cp genomes, mononucleotide repeats, ranging from 31 to 36, emerged as the most prevalent SSR type. Dinucleotide repeats numbered between 10 and 11, trinucleotide repeats ranged from 3 to 5, tetranucleotide repeats varied from 7 to 8, pentanucleotide repeats oscillated between 2 and 3, and hexanucleotide repeats manifested a singular instance in *P. odoratum* (Fig. 4, Table S1). Strikingly, SSRs enriched with of A/T outpaced those harboring G or C, with A/T repeats constituting 35 (57.4%), 31 (57.4%), 36 (60.0%), and 33 (55.0%) in *P. filipes*, *P. cyrtonema*, *P. zanlanscianense* and *P. odoratum*, respectively. An intriguing observation surfaced—the cp genomes of *P. cyrtonema* and *P. zanlanscianense* lacked C/G SSR repeats (Fig. 4a). Moreover, distinctions in SSR counts and types were apparent across the cp genomes of the four plants. *P. odoratum* showcased additional types and repetition units of SSRs, exemplified by the distinctive CCT/TAT trinucleotide and the ATAGTA sequence of hexanucleotide sequence. The majority of SSRs primarily occupied the LSC and SSC regions, as opposed to the IR region (Fig. 4a, Table S1). Remarkably consistent findings characterised the four *Polygonatum* plants in GenBank sharing the same species names. Notably, SSR counts ranged from 56 to 61, featuring mononucleotide to pentanucleotide repeats and a conspicuous absence of hexanucleotide repeats. These plants also lacked the CCT/TAT motif, while and the count of other SSR types maintained consistency or differed by a more one instance. These observations underscore the relative stability of SSRs in the genus *Polygonatum*, asserting their resilience against environmental influences and affirming their utility as a potent tool for species differentiation. In sum, the presented findings hold substantial implications for comprehending intrageneric and intergeneric relationship within, illuminating phylogenetic connections, and suggesting the potential for SSR-specific markers to fuel genetic diversity analysis and classification within the *Polygonatum* genus*.*

Diving into the realm of oligonucleotide repeats through meticulous analysis using REPuter software, a quartet of repeat sequences unveiled themselves within the cp genomes of *Polygonatum*. This collection encompassed palindrome, forward, reverse, and complementary repeats, all of which exceeded a length of 30 bp (Fig. 4b). Within the realm of *P. filipes*, a compendium 35 long repeat sequences were discerned, embracing 17 palindromic, 17 forward, and 1 reverse repetitions. Notably, *P. cyrtonema* showcased the most prolificity, tallying up to 49 repeats comprising 28 palindromic, 16 forward, and 5 reverse iterations. *P. zanlanscianense* displayed 22 palindromic, 22 forward, 3 reverse, and 1 complementary repeat, while *P. odoratum* boasted 24 palindromic, 22 forward, and 1 reverse repeat. Of particular note was the exclusive presence of complementary repeats in *P. zanlanscianense*, a feature absent in the other three species (Fig. 4b). Predominantly, long repeat sequences spanned the spectrum of 30 to 39 bp in length, with palindromic repeats emerging as the most profuse category. It is noteworthy that the scrutiny extended to the oligonucleotide repeat analysis of four *Polygonatum* cp genomes with matching species names in GenBank. Predictably, *P. filipes* (MZ150843) exhibited the scantest collection of long repeat sequences, while a discerning distinction arose in *P. zanlanscianense* (MW800891), which lacked complementary repeats. The in-depth exploration of repeats imparts a guiding compass for unraveling the tapestry of genetic diversity and population dynamics among species.

### IR expansion and contraction analysis

**Figure 5:**
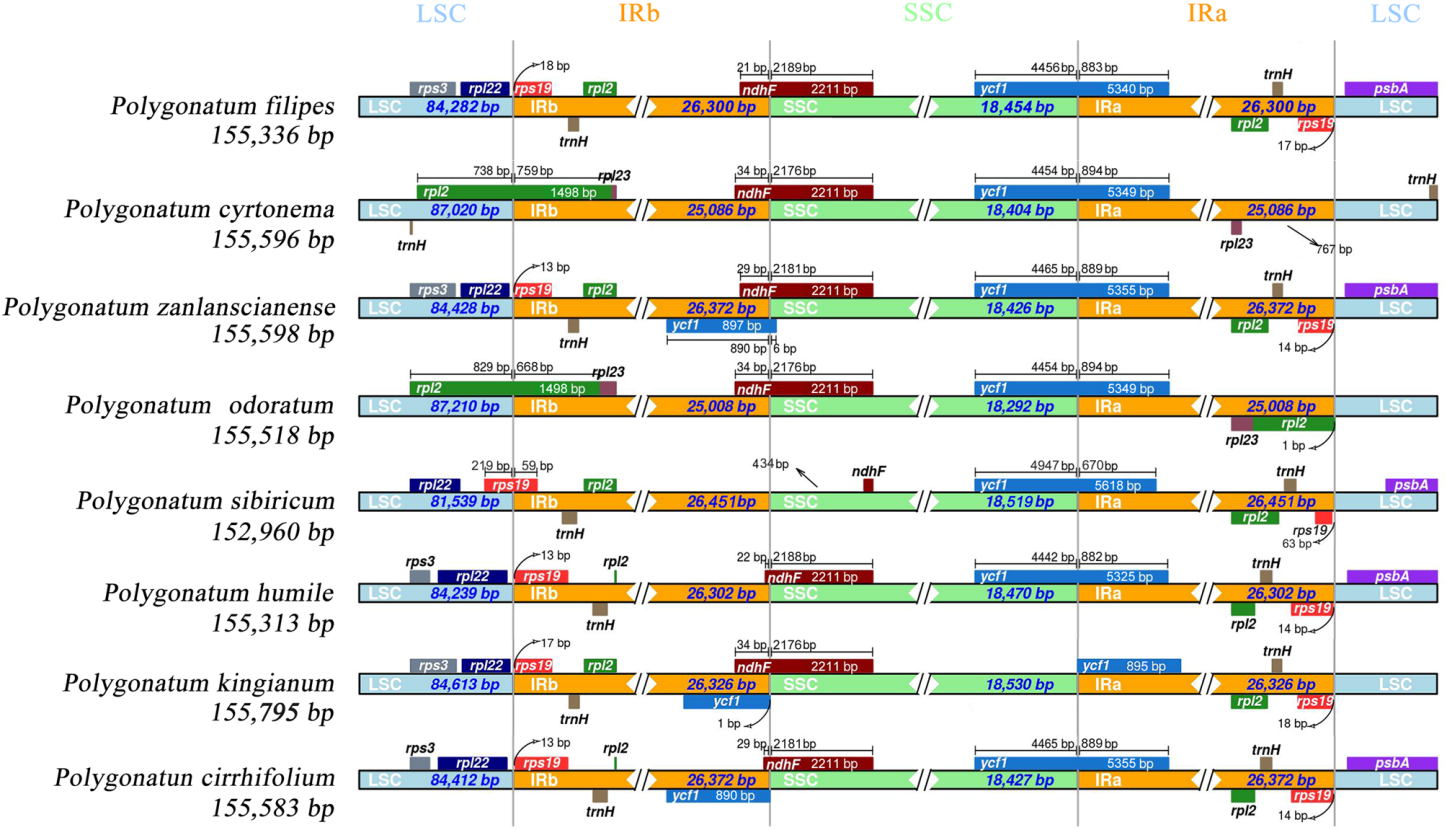
A comparison of the border distances between adjacent genes and the junction of the LSC, SSC and IRs regions in the eight chloroplast genomes of *Polygonatum*.

The ebb and flow of the IR region, often characterized by expansions and contractions, stands as a frequent phenomenon in genome evolution, assumedly driving shifts in the lengths of diverse cp genomes. This phenomenon doubles as a potent tool for dissecting study phylogenetic relationships and taxonomic classifications^27^. In our endeavor, we diligently scrutinized the boundaries of LSC/IRb/SSC/IRa regions across four *Polygonatum* species and their closely aligned counterparts (inclusive of *P. sibiricum*, *P. humile*, *P. kingianum* and *P. cirrhifolium*). This analytical pursuit aimed at pinpointing their distinctive and shared cp genomic characteristics, detailed insights of which are elucidated in Fig. 5. Within the bounds of all *Polygonatum* plants, IR regions exhibited a similar length, spanning from 25,008 bp in *P. odoratum* to 26,451 bp in *P. sibiricum*. Although changes in the length of the IR region remained marginal, discernible variances between IR region expansions and contractions emerged. The *rps19* gene, spanning 18 bp, 13 bp, 13 bp, 17 bp and 13 bp from the LSC-IRb boundary in *P. filipes*, *P. zanlanscianense*, *P. humile*, *P. kingianum* and *P. cirrhifolium*, respectively, encapsulated the IRb region entirely. Notably, *P. sibiricum* diverged from this trend, positioning the LSC-IRb boundary within the *rps19* gene, enveloping 59 bp into the IRb region. Conversely, the *rpl2* gene, spanning 759 bp and 668 bp distances to the IRb region, was predicted within the LSC-IRb region for *P. cyrtonema* and *P. odoratum*, respectively (Fig. 5). Occupying the IRb-SSC junction region, excluding *P. sibiricum*, seven other plants nestled within the *ndhF* gene's coding region, bridging the IRb and SSC regions. In this context, the *ndhF* gene's length in the IRb region fluctuated from 21 bp in *P. filipes* to 34 bp in *P. cyrtonema*, *P. odoratum* and *P. kingianum*. *P. sibiricum*'s *ndhF* gene was positioned wholly within the SSC region, separated by 434 bp from the IRb-SSC junction. With the exception of *P. kingianum*, the *ycf1* gene, acted as the SSC-IRa junction nexus for the other plants, its distance from the IRa region spanning 670–894 bp. *P. kingianum* diverged in that its *ycf1* gene was exclusively located within the IRa region. Meanwhile, a substantial portion of the *trnH* gene was predominantly allocated within in the IRa region throughout the cp genomes of *Polygonatum* species (Fig. 5). It is noteworthy that the *P. cyrtonema* (MZ029094) entry from GenBank, featured an LSC-IRb boundary adorned with the *rps19* gene, stretching 150 bp into the IRb region^16^. This scenario echoed the arrangement discerned in *P. sibiricum*, whereas in most other species of *Polygonatum*, the *rps1*9 gene resided entirely within the IRb region. Notably, our sequencing results for *P. cyrtonema* indicated an absence of the *rps19* gene, which could be attributed to the extended length of the *rpl2* gene (1498 bp), bridging the LSC and IRb regions. In contrast, the *rpl2* gene in *P. cyrtonema* (MZ029094) was truncated, confined solely within the LSC region. The shifts within the four boundary regions of *Polygonatum* species’ cp genomes culminated in changes in genome length and the comprehensive genome sequence. Of particular intrigue, the *ψycf1* pseudogene found its abode within the IRb region of in the *P. kingianum* and *P. cirrhifolium* cp genomes. The previously mentioned *ycf1* gene, situated at the juncture of SSC and IRa regions in *P. sibiricum*, was similarly rendered a pseudogene. The distribution and sequence of these genes across the four regions in the *Polygonatum* genus echoed the trends observed in *Heteropolygonatum*, *Disporopsis* and *Maianthemum*^16^. Noteworthy was the *ycf1* gene, typically situated at the SSC and IRa region intersection, with a small subset of plants being exclusively IRa-bound, an observation that held true for both monocotyledons and dicotyledons^28^^,^^29^.

### Identification and analysis of divergence regions and the establishment of barcodes

**Figure 6:**
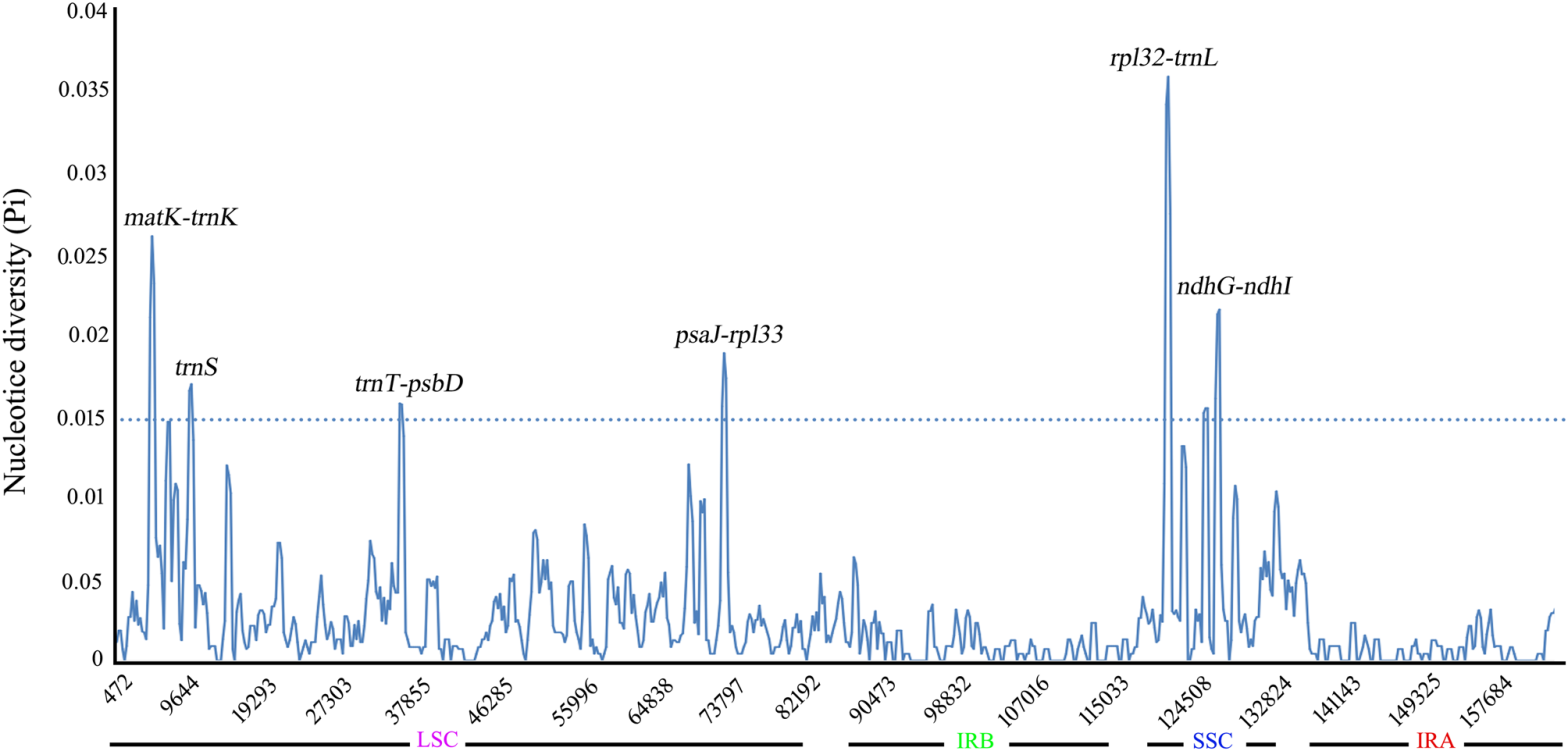
Nucleotide diversity (Pi) analysis of chloroplast genomes in *Polygonatum* species with 600 bp sliding window length and 200 bp step size.

**Figure 7:**
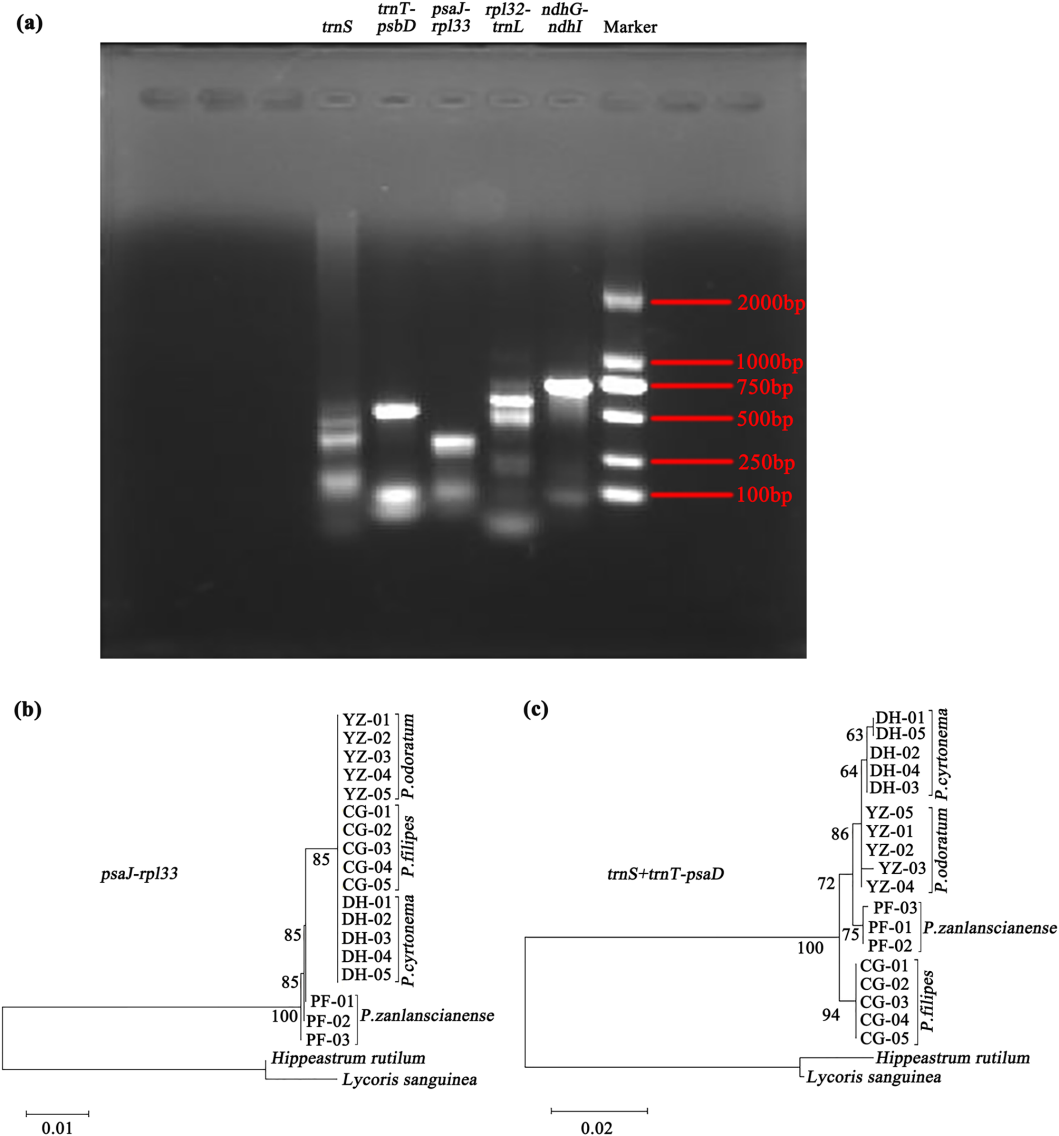
(**a**) Five pairs of primers were detected by agarose gel electrophoresis and from left to right were *trnS*, *trnT-psbD*, *psaJ-rpl33*, *rpl32-trnL* and *ndhG-ndhI*, respectively. *P. cyrtonema* of the plant was used for PCR amplification in this figure. We analyzed the evolution and development of the four *Polygonatum* species. MEGA v11 was used for maximum likelihood (ML) analysis to construct a phylogenetic tree, which was built with 1000 bootstrap replications on the Kimura 2-parameter model. (**b**) The analysis of the *psaJ-rpl33* sequence fragment (**c**) The analysis of *trnS* + *trnT-psbD* sequence fragments.

To gauge the extent of sequence divergence, multiple comparisons were carried out on the complete cp genomes of the *Polygonatum* species. The DnaSP software was employed to calculate nucleotide diversity (Pi). Within this context, six hypervariable regions emerged, characterized by Pi values surpassing 0.015. These regions included *matK-trnK*, *trnS*, *trnT-psbD*, *psaJ-rpl33*, *rpl32-trnL*, and *ndhG-ndhI* (Fig. 6). These high-Pi regions are potential divergence sites within the complete cp genomes of the four *Polygonatum* species. Among these regions, the LSC region housed four mutational hotspots (*matK-trnK*, *trnS*, *trnT-psbD*, *psaJ-rpl33*), while in the SSC region contained two (*rpl32-trnL*, *ndhG-ndhI*). Remarkably, no high mutation sites were found within the IR regions, solidifying the notion that the IR regions remained highly conserved in the cp genomes of *Polygonatum* plants' cp genomes. These six sequences harboring high Pi values could be utilized as DNA markers to unveil the genetic divergence among *Polygonatum* taxa. Leveraging the conserved regions surrounding these variable regions of the mutational hotspots, we proceeded to design primers for molecular markers. During this primer design process, we discovered that the GC content of the primer designated for the *matK-trnK* sequence was notably lower than the recommended 40%, resulting in an excessively low final software score. This shortcoming could potentially hinder the amplification of samples, prompting the exclusion of this sequence from primer design considerations. Ultimately, we designed five pairs of PCR primers (*trnS*, *trnT-psbD*, *psaJ-rpl33*, *rpl32-trnL*, and *ndhG-ndhI*) were designed as potential molecular markers (Table 6). Agarose gel electrophoresis confirmed that *rpl32-trnL* and *ndhG-ndhI* exhibited prominent main bands (Fig. 7a). However, upon PCR amplification sequencing, it was revealed of the samples showed that *rpl32-trnL* and *ndhG-ndhI* often exhibited double peaks and primer dimers, lacking practical application significance. As an alternative approach, 18 samples from the four *Polygonatum* species were amplified and sequenced using *trnS*, *trnT-psbD* and *psaJ-rpl33* barcode universal primers. The amplification products generated by the *trnS* primer ranged from 395 to 405 bp, while the *trnT-psaD* primer yielded products spanning 445 bp to 471 bp. For the *psaJ-rpl33* primer, the amplified fragments ranged from 245 to 272 bp. Notably, the length and GC content of *P. odoratum* samples amplified with these three primers were consistent, suggesting stable genetic traits within the *P. odoratum* species (Table 7). Phylogenetic analysis demonstrated that the *psaJ-rpl33* barcode was capable of distinguishing *P. zanlanscianense*, with samples from the other three *Polygonatum* species clustering together. While the *trnS* and *trnT-psbD* barcodes individually were less effective in distinguishing the four species, the combination of *trnT-psaD* and *trnS* barcodes divided the four *Polygonatum* species into separate branches, implying that the *trnS* + *trnT-psbD* barcodes were able to differentiate the four species of *Polygonatum*, effectively (Fig. 7b,c). This approach of combining DNA barcodes for plant identification echoes similar successes in the research of other plant families, such as Lauraceae, *Astragalus L*. plants, and various herbaceous woody plant species^30–32^. Therefore, the *psaJ-rpl33* and *trnS* + *trnT-psbD* molecular markers exhibit promising potential for species identification and population genetic studies of *Polygonatum* plants.

**Table 6:** PCR primers designed according to the mutational hotspots within eight *Polygonatum* species.

Mutational hotspots	PCR primers
*trnS*	F:ATCAGAGGGAACGGAGAGAGAGGG
	R:GTACTTAACCAGGCCGGGGGAATG
*trnT-psbD*	F: CTCTTCTTGTTAATGGATGCTGCAT
	R: AACACTTCATCTCATTTCTAGGGCA
*psaJ-rpl33*	F: GTTTGGGTCTTTAGCGGGTCTAT
	R: CCCCCCTTTCTATTTCTGTTCTC
*rpl32-trnL*	F: AGAACTAGCTGCTATGATATG
	R: GTCGAAACAGAAACTAAGAAT
*ndhG-ndhI*	F: ACTCCATCTCCAACAGTCCAAAAA
	R: TCAAAATAGACAAGGATAACCCCC

**Table 7:** Sequence analysis of *Polygonatum* samples basing on three DNA barcodes.

Species/accession number	Sample number	Sample source	*trnS*			*trnT-psaD*			*psaJ-rpl33*		
			Length (bp)	GC content (%)	GenBank accession No	Length (bp)	GC content (%)	GenBank accession No	Length (bp)	GC content (%)	GenBank accession No
*P. filipes* MZ571521	CG-01	Suichang County, Lishui, Zhejiang 28°52′N, 119°11′E	402	25.62	OP610071	450	31.78	OP610101	265	27.92	OP610089
	CG-02				OP610072	445	31.69	OP610102			OP610090
	CG-03				OP610073	468	32.48	OP610103			OP610091
	CG-04				OP610074	445	31.69	OP610104			OP610092
	CG-05				OP610075	450	31.78	OP610105			OP610093
*P. cyrtonema* MZ579646	DH-01	Suichang County, Lishui, Zhejiang 28°52′N, 119°11′E	395	25.32	OP610070	471	30.57	OP610106	272	26.84	OP610094
	DH-02		403	25.56	OP610076	470	30.43	OP610107	265	27.92	OP610095
	DH-03				OP610077			OP610108			OP610096
	DH-04				OP610078	469	30.49	OP610109	272	26.84	OP610097
	DH-05		395	25.32	OP610115	471	30.57	OP610120			OP610098
*P. zanlanscianense* MZ568930	PF-01	Suichang County, Lishui, Zhejiang 28°52′N, 119°11′E	405	25.68	OP610067	446	31.61	OP610068	265	27.55	OP610069
	PF-02				OP610116			OP610118			OP610099
	PF-03				OP610117	454	31.28	OP610119			OP610100
*P. odoratum* MZ666387	YZ-01	Bozhou, Anhui 33°86′N, 115°78′E	403	25.56	OP610079	469	30.49	OP610110	245	30.20	OP610084
	YZ-02				OP610080			OP610111			OP610085
	YZ-03				OP610081			OP610112			OP610086
	YZ-04				OP610082			OP610113			OP610087
	YZ-05				OP610083			OP610114			OP610088
*Lycoris sanguinea*		GenBank	369	27.64	NC_047453	456	31.58	NC_047453	215	33.02	NC_047453
*Hippeastrum rutilum*		GenBank	365	27.40	NC_053356	483	30.64	NC_053356	212	33.02	NC_053356

### Phylogenetic analysis

**Figure 8:**
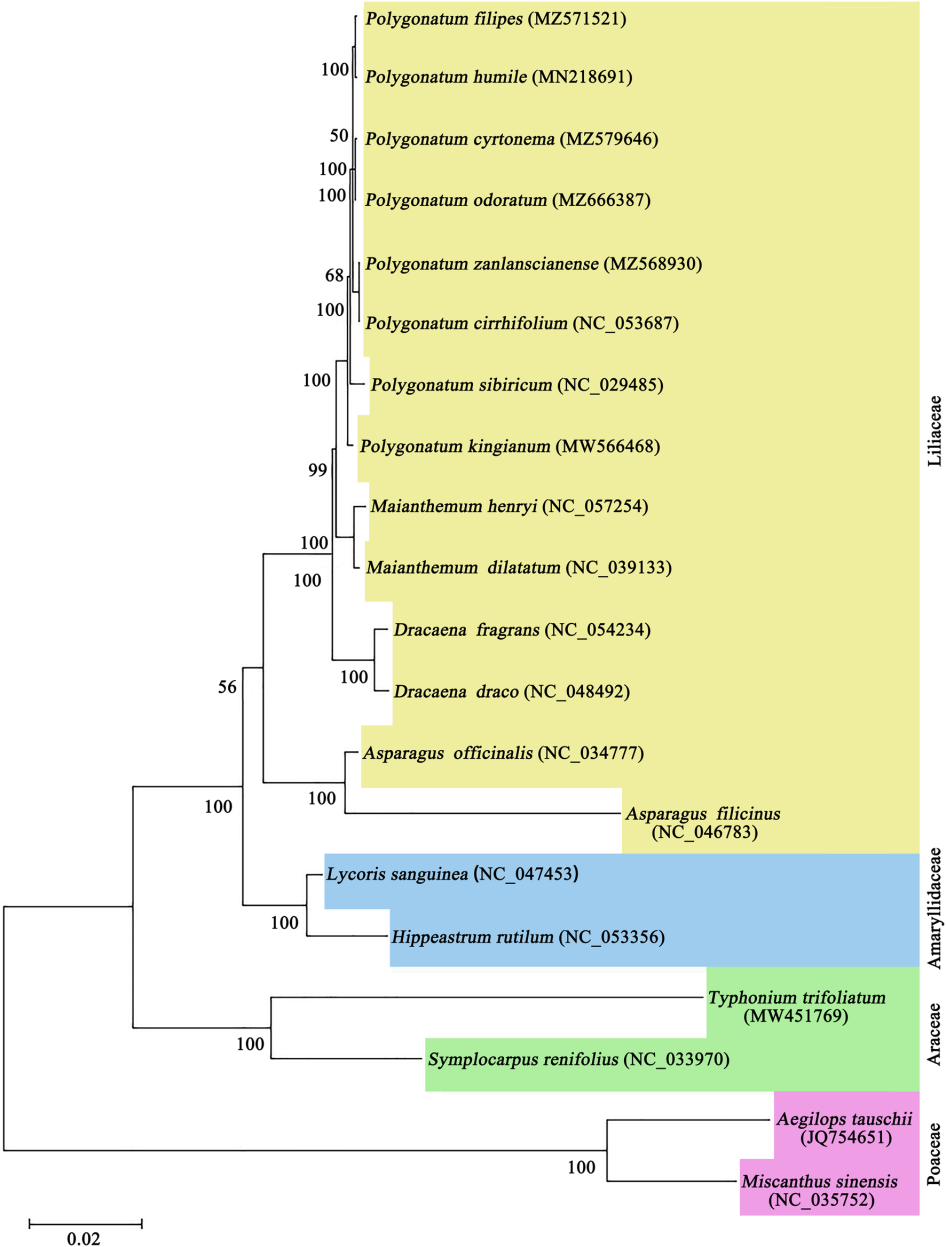
The phylogenetic tree was constructed by the Maximum likelihood method based on the complete chloroplast genomes of 20 species, with *Aegilops tauschii* and *Miscanthus sinensis* as the outgroup plants.

Chloroplast genomes are valuable resources for unveiling phylogenetic relationships among various plant taxa. They have been extensively used for phylogenetic analyses across different plant groups^33^. In order to establish the phylogenetic position of the *Polygonatum* genus, a phylogenetic tree was constructed using complete cp genome sequences. This was achieved through the maximum likelihood (ML) method, and involved the inclusion of 14 species from the Liliaceae family, 2 species from Amaryllidaceae, 2 species from Araceae and 2 species from Poaceae. Two outgroup species, *Aegilops tauschii* and *Miscanthus sinensis* were selected as outgroups for this analysis. The resulting phylogenetic tree (Fig. 8) exhibited strong statistical support for most nodes, barring a few terminal nodes. The analysis revealed that all the species formed four major evolutionary branches, aligning with the corresponding families. The eight *Polygonatum* plants (*P. filipes*, *P. cyrtonema*, *P. zanlanscianens*, *P. odoratum*, *P. sibiricum*, *P. humile*, *P. kingianum*) and *P. cirrhifolium* constituted a stable monophyletic group, indicative of their close genetic relationship. However, it was evident that plants belonging to the same series were not consistently placed within the same branch, implying some divergence between species of the same series and those from different series (Fig. 8). Additionally, the *Maianthemum* genus formed a branch that grouped with the *Polygonatum* genus, displaying robust with high statistical support. This outcome underscored the close relationship between the *Maianthemum* and *Polygonatum* genera within the *Polygonateae* group. Similarly, species from the *Polygonateae* and *Dracaeneae* tribes formed a single branch, reflecting their shared affiliation within Liliaceae family. The comprehensive cp genome data from the four *Polygonatum* species contributed significant insights into the phylogenetic relationships and species classification within the Liliaceae family.

## Discussion

Our investigations have successfully elucidated the entire cp genomes of four plant specimens belonging to the *Polygonatum* genus, followed by a meticulous analysis of their intrinsic traits. This comprehensive scrutiny encompassed an exploration of codon usage biases, RNA editing sites, and repetitive sequences inherent to the complete cp genomes of the four distinct *Polygonatum* species. Furthermore, a phylogenetic tree was meticulously constructed, encompassing species that share a kinship. The outcomes of these analyses have furnished fundamental groundwork, priming the stage for further in-depth inquiries into the taxonomy and phylogenetic relationships intrinsic to the *Polygonatum* genus.

Since the inception of the *Polygonatum* genus, the classification, species identification, and overall characterization of entities nested within this genus have presented formidable enigmas demanding resolution^34^. An array of *Polygonatum* variants, characterised by their alternate leaves and cylindrical rhizomes, have historically been erroneously classified as *P. odoratum*, consequently rendering the commercial origins thereof quite intricate. The attendant intricacies have contributed to confounding uncertainties in discriminating between varieties of *P. odoratum*. Adding to the intricacy, the inconspicuous morphological distinctions among original *Polygonatum* plants, coupled with their overlapping geographical distribution, have compounded the intricacies within this taxonomic group^35^. Notably, a dedicated exploration into the the phytochemical composition of *P. prattii* was executed. The results of chemotaxonomic analyses have revealed compelling indications that *Polygonatum* exhibits close affinities with *Liriope*, *Disporopsis* and *Ophiopogon*, particularly in regard to the composition of steroids and homoisoflavanones^36^. The confluence of diverse origins and intricate chemical configurations has consequently posed substantial challenges to the accurate classification and precise identification of *Polygonatum* plants. Consequently, the imperative to differentially distinguish the *Polygonatum* genus from other rhizomatous species gains paramount importance, particularly in safeguarding product integrity and consumer well-being. In light of the inefficacies intrinsic to conventional molecular identification methodologies, it is manifestly vital to advance more fficacious means of distinguishing *Polygonatum* plants.

Codons, constituting sequential units of genetic information transference within organisms, play an instrumental role in comprehending biological evolution and phylogenesis. The examination of codon usage bias and RNA editing assumes paramount significance in this regard. An exploration into codon usage patterns within *Lonicera heckrottii* has illuminated the fact that preferential utilisation of codons in cp genes is substantially influenced by mutation pressures, natural selection dynamics, and the prevailing GC content^37^. Across angiosperms, discernible preferences exist for most amino acid codons, although this phenomenon is not observed in the cases of methionine and tryptophan. The plant specimens under our scrutiny conform to this pattern, with 31 codons exhibiting RSCU values surpassing unity and an equal count of 31 codons showcasing RSCU values below unity. Notably, AGA attains the zenith with a value of 1.93, while CGC plumbs the nadir with a value of 0.30 (Fig. 2, Table 3). Evidently, among the 31 codons boasting RSCU values exceeding 1, a substantial majority, 29 to be precise, culminate with A or U, while a corresponding 28 codons displaying RSCU values beneath1 culminate with G or C. This trend underscores the predilection within *Polygonatum* plants towards the adoption of synonymous codons concluding with a third base of A or U, paralleling observations in *Stephania tetrandra*^18^ and certain other angiosperms^38^^,^^39^. The preponderance of A/U-rich positions evidently contributes significantly to the emergence of codon usage bias^40^. Furthermore, the GC content within the third position, particularly susceptible to mutations, registers lower values compared to the two preceding positions across all codons^41^. Moreover, it is noteworthy that the *rps19* and *rpl2* genes within the four examined plants set forth with initiation codons GTG and ATA, respectively, as opposed to the conventional ATG. Notably, unconventional initiation codons such as TAT and CTA have been identified in *P. filipes* and *P. cyrtonema*, respectively. Morton et al.^42^ postulated that the interplay of selection pressures and mutational dynamics potentially exerts influence upon codon preferences within the chloroplast genome. Delving into the intricacies of codon preference augments our comprehension of gene expression modalities and molecular evolutionary trajectories specific to the *Polygonatum* genus.

The assortment of RNA editing mechanisms potentially contributes significantly to the evolutionary trajectory of RNA editing in plants^43^. In our investigation, we have ascertained the presence of 59–64 potential RNA editing sites across 22 protein-coding genes nested within the chloroplast genomes of four distinct *Polygonatum* species (Fig. 3). These sites of RNA editing engendered modifications leading to ten diverse amino acid conversions (Table 4). Noteworthy among these is the instance of LPA66, which was implicated in the editing of the *psbF* gene; the consequential alterations in amino acids, due to the absence of editing, hindered the efficient assembly of Photosystem II (PSII) complexes^44^. In concurrence with our findings, Sasaki T et al. highlighted the occurrence of a C residue within *N. tabacum* and *N. sylvestris*, earmarked for editing, yet lacking the corresponding editing activity within *N. tomentosiformis*^45^, aligning somewhat with our observations. Delving into the intricacies of RNA editing sites within the chloroplast genomes stands as a pivotal pursuit for comprehending the accuracy of translational processes and the propensity for genetic mutations^46^. For instance, our analysis unveiled the absence of RNA editing sites within the *rpl2* gene of *P. zanlanscianense* and the *ycf3* gene within *P. cyrtonema* and *P. odoratum* (Fig. 3). Remarkably, and the p*afI* gene was found in close proximity to*ycf3*. This phenomenon may stem from shifts in RNA editing sites, engendering divergent translations and gene mutability. Nevertheless, the veracity of this supposition necessitates further inquiry. The assessment of Ka/Ks values across protein-coding genes adorned with RNA editing sites extends insights into functional diversity, structural alterations, and species evolution. The Ka/Ks ratio has frequently served as a barometer for discerning if genes encoding proteins are subject to selection pressure and has found wide application as an effective gauge reflecting species' adaptive evolution rates and the impact of positive selection pressure^47^. A cursory examination of Table 5 reveals that most genes experienced negative selection pressure, aligning with conservative evolutionary trends. Notably, only two genes (*accD* and *rpl2*) displayed Ka/Ks values surpassing unity. It is often that genes under positive selection are a numerical minority, however, the regions conferring functional attributes of a gene are pivotal in influencing its functional impact. In instances where positive selection transpires at specific sites, repercussions on its function can be profound. For instance, within the genomes of two *Trichoderma hamatum* strains, the gene *YYH13* under positive selection impacted the synthesis and extracellular secretion of antibiotics, while the positive selection-afflicted gene *YYH16* focused on the metabolic pathway of cellular endocrinology, possibly contributing to trait differences between the two strains^48^. Within the chloroplast genome of *Chrysosplenium*, genes involved in photosynthesis—*matK* and *ycf2*—evinced strong positive selection, a response that was deemed adaptive to their humid and shaded habitat^49^. In a deviation from the prevailing notion that *matK* ranks among the fastest-evolving genes within the chloroplast genome^50^, our study has revealed two remarkable cases of positively selected genes. In plants, the regulation of synthesis efficiency, a facet inherently linked to ribosomal function, is a countermeasure for adapting to dynamic developmental stages and ever-changing environments, bearing profound significance for growth. This underscores the relevance of our observation concerning the robust positive selection of the *rpl2* gene within *Polygonatum* plants, potentially attributed to its role as a ribosomal protein. Similarly, studies on ribosomal protein mutations in *Arabidopsis thaliana* encompassed embryonic, leaf, root development, and stress responses^51^. In the same vein, the *accD* gene, despite its elusive function, garnered attention due to its elevated Ka/Ks value. This obscurity may nonetheless provide pivotal insights for future investigations into the adaptive evolution of the *Polygonatum* genus. Evidently, the *rpl2* and *accD* genes stand forth as pivotal candidates subject to vigorous positive selection, significantly shaping the evolutionary trajectory of the *Polygonatum* genus.

Simple sequence repeats (SSRs), known for their pronounced polymorphism, can be harnessed as molecular markers to facilitate species identification and the analysis of phylogenetic relationships. Insightful statistical analysis coupled with cluster examination of SSR attributes has illuminated the potential of Cyatheaceae cp SSR distributions to convey informative phylogenetic signals at the genus level^52^. This methodology was similarly employed in exploring the SSR distribution nuances within *Cypressus*, unveiling a notable congruence in the distribution patterns between *Cupressus* and *Hesperocyparis*^53^. Contrarily, substantial deviations in SSR types among genera were observed within Dryopteridaceae^54^. In the context of our current investigation, an enumeration of 54–61 SSRs was undertaken across the cp genomes of four distinct *Polygonatum* species. Intriguingly, mononucleotide repeats emerged as the most prevalent SSR type. Particularly, it was observed that mononucleotide repeat A/T assumed eminence as the most abundant, closely trailed by the dinucleotide repeat AT/TA (Fig. 4a), a concurrence that mirrors earlier findings^55^^,^^56^. This encompassing knowledge reservoir concerning SSR patterns holds the potential to serve as a valuable resource in the quest for molecular markers dedicated to *Polygonatum* species identification, genetic diversity assessment, and the elucidation of phylogenetic relationships.

DNA barcoding, a potent technique for molecular identification, has yielded remarkable outcomes across diverse domains, including animals and plants, thereby substantially catalysing the advancement of molecular phylogenetics. Within the context of plant genomes, regions of heightened variability within the cp genome have emerged as potentially robust DNA barcodes, offering precise identification of closely related species and enabling the analysis of phylogenetic relationships. This propensity for employing cp genome regions as DNA barcodes is exemplified by Song et al., who identified six variable markers and designated the *trnT-trnL* and *ycf1b* genes, boasting elevated success rates in identification, as cp DNA barcodes specific to *Styrax*^57^. These sequences have significantly enriched resources for species determination, phyletic evolution insights, and population genetics research. Our investigation has unveiled six hyper-variable regions, encompassing five intergenic regions (*matK-trnK*, *trnT-psbD*, *psaJ-rpl33*, *rpl32-trnL* and *ndhG-ndhI*), and a tRNA gene (*trnS*). Notably, the Pi values within the IR regions of *Polygonatum* plants were conspicuously lower compared to those within the SSC and LSC regions, aligning with previous findings in our study of various plant species^58^. This characteristic holds true across the angiosperms, where intergenic spacers generally exhibit greater sequence variation. The segments demonstrating elevated Pi values were considered divergence-prone zones within the complete cp genomes of the four *Polygonatum* species. Most notably, the *rpl32-trnL* region exhibited the highest Pi values, followed by *matK-trnK*. Interestingly, *matK* has also been advocated as a fundamental universal DNA barcode in *Diospyros* plants^59^. However, both of these regions presented limitations in terms of practical applicability concerning primer design and the sequencing of PCR-amplified samples. In contrast, *trnS*, *trnT-psbD*, and *psaJ-rpl33* segments were robustly amplified and sequenced across 18 samples spanning the four plant species (Table 7). The utilization of cp genome-based DNA barcoding stands as a promising avenue for discerning distinct plant species. This is exemplified by the application of a DNA barcoding method founded upon ITS2 and *psbA-trnH* to identify 39 samples of *Polygonati rhizoma* from southern China, effectively and accurately distinguishing *Polygonati rhizoma* from morphologically similar species with the same rhizome features^11^. Presently, DNA barcoding investigations within the *Polygonatum* genus tend to concentrate on ITS, *matK* and *psbA-trnH*. However, these three sequences face the challenge of low identification efficiency^11,60,61^. Consequently, there is an urgency to identify other highly variable regions of the genome and design corresponding primers to enable the differentiation of *Polygonatum* species at the genus level. Our study endeavoured to amplify and sequence samples employing five pairs of designed primers. The resultant phylogenetic tree outcomes underscored the discriminatory power of *psaJ-rpl33* in distinguishing *P. zanlanscianense* from the remaining three *Polygonatum* species. Similarly, the molecular phylogeny constructed based on *trnS* + *trnT-psbD* barcodes effectively delineated the *Polygonatum* genus into four distinct branches (Fig. 7). The *psaJ-rpl33* and *trnS* + *trnT-psaD* molecular markers demonstrated robust identification capabilities within the classification and molecular phylogeny of the *Polygonatum* genus, introducing novel avenues that have yet to be explored in the context of DNA barcoding of *Polygonatum* medicinal plants. These discoveries expand the repertoire of potential DNA barcodes within the *Polygonatum* genus, while also augmenting the available resources for the identification and elucidation of phylogenetic relationships among *Polygonatum* plants.

Initially, the placement of the *Polygonatum* genus was within the Asparagaceae family, later being reclassified under the Liliaceae family as per the APG IV system. As per the Flora of China (1978), in terms of the type of phyllotaxis and the length of the perianth tube, *Polygonatum* was divided into eight sub-groups based on phyllotaxis type and perianth tube length, including Ser. *Bracteata*, Ser. *Alternifolia*, Ser. *Kingiana*, Ser. *Hookeriana*, Ser. *Punctala*, Ser. *Alte-lobata*, Ser. *Oppositifolia* and Ser. *Verticillata*. Despite the complexities inherent to the identification and classification of the *Polygonatum* genus, leveraging phylogenetic analysis using the cp genome often proves advantageous in elucidating deep-seated relationships between plant lineages^62^. Given its substantial species count and morphological similarities, the *Polygonatum* genus poses a taxonomic challenge. Wang et al. conducted a study wherein phylogenetic analysis of 88 cp genomes robustly endorsed the monophyly of *Polygonatum*, with *HeteroPolygonatum* emerging as its sister group, albeit with a preceding divergence time compared to *Disporopsis*, *Maianthemum* and *Disporum*^16^. In our investigation, the ML tree results portrayed that the eight *Polygonatum* species coalesced into a monophyletic clade within the evolutionary framework of Liliaceae, forming a close alliance with *Maianthemum*, *Dracaeneae* and *Asparageae*. In this phylogenetic tree our findings closely resembled the Flora of China's classification, albeit with slight deviations. Notably, *P. filipes*, *P. cyrtonema*, *P. odoratum* and *P. humile*, constituents of Ser. *Alternifolia Baker*, formed two smaller branches, ultimately reinforcing the overall four-cluster structure of the phylogenetic tree. Intriguingly, *P. sibiricum*, classified under Ser. *Verticillata*, diverged from the *P. zanlanscianens* and *P. cirrhifolium*, displaying lower support with the branch of* P. cyrtonema* and *P. odoratum* branch. This divergence could be attributed to evolutionary shifts and accompanying morphological alterations within *P. sibiricum* or potentially driven by geographical isolation from *P. zanlanscianens* and *P. cirrhifolium*. Li et al. posited that the interspecific clustering of 38 *Lilium* species harmonised with Comber’s the morphological classification system^63^, while acknowledging the need for reclassifying lily varieties that have historically been contentious^64^. The delineation of boundaries between Liliaceae genera remains a subject of controversy, chiefly stemming from the subjectivity intrinsic to traditional morphological classification methods and the influence of environmental factors on plant morphological traits. Significantly, the *P. kingianum*, affiliated with Ser. *Kingiana*, established an independent branch, aligning with extant research classifications. The closely similar branch lengths among the eight *Polygonatum* species corroborated their close genetic relationships. Overall, the evolutionary relationships deduced from our study generally aligned with prior investigations, affirming the monophyly of *Polygonatum* and its relationship with other Liliaceae genera^16,65^. This underscores the progress facilitated by comprehensive cp genome analysis within certain phylogenetic lineages. Additionally, within a larger clade, the Liliaceae genera *Maianthemum*, *Dracaena* and *Asparageae* exhibited closer associations with *Polygonatum*. Concurrently, species from three other families formed distinct evolutionary branches, corresponding to their respective lineages. The evolutionary tree results vindicated the rationale behind the existing *Polygonatum* classification system based on established phylogenetic relationships. However, further evidence is imperative to unravel evolutionary ties and internal divisions. Notably, the cp genome sequence data furnishes a theoretical foundation for informed species classification and the elucidation of phylogenetic relationships within the *Polygonatum* genus.

This article unveils notable distinctions within the protein-coding genes of complete cp sequences from *P. cyrtonema* and *P. odoratum* compared to other *Polygonatum* species. Specifically, the absence of the ribosomal protein gene *rps19* in the LSC region, a phenomenon also identified in *Ophiopogon* and *Dracaena*, marks a significant variation^66^^,^^67^. Moreover, the *ycf3* and *ycf4* genes absent in *P. cyrtonema* and *P. odoratum*, while the *pafI* and *pafII* genes appear in their close vicinity, respectively. Intriguingly, this gene loss pattern contrasts with previous analyses of four *Polygonatum* plants sharing the same species name in GenBank, wherein *ycf3* and *ycf4* were not deleted. Of note, *ycf3* and *ycf4* encode proteins of enigmatic function, mirroring the uncertain roles of *pafI* and *pafII*. These aforementioned genes hold potential as indicators for investigating the evolutionary trajectory and phylogenetic, connections among *Polygonatum* plants. A noteworthy finding pertains to the *pbf1* gene, observed exclusively within the four sequenced species, with other *Polygonatum* plants featuring *psbn* instead^24^. This contrast was also evident among the four *Polygonatum* plants with the same species designation in GenBank. Intriguingly, the *pbf1* gene isn't restricted to the *Polygonatum* genus; it is also found in *Ophiopogon japonicus* (NC_049869) of the *Ophiopogon* genus. However, the mechanism governing the emergence and loss of these protein-coding genes in the *Polygonatum* genus remains shrouded, as does the question of whether these genes' functions can be complemented by proteins encoded in the nuclear genome. Shifting focus, the cp genomes of the four examined species showcase gene diversity. Notably, the *ndhK* gene, an enzyme located in the inner mitochondrial membrane facilitating electron transfer from NADH to coenzyme Q, exhibited variations. *P. odoratum*'s *ndhK* reading frame spans 770 codons, shorter than the 881-codon length in the other three Polygonatum species. Prior studies indicate that *ndhK* encounters growth limitations under low CO_2_ or heterotrophic conditions, suggesting its pivotal role in CO_2_ absorption^68^. High light conditions hindered the growth of *ndhK* gene deletion mutants and correlated physiological parameters^69^. This might suggest that the discrepancy in the *ndhK* gene might be attributed to *P. odoratum*'s affinity for humid environments, distinct from the shaded forest or hillside preferences of the other three *Polygonatum* species. The *ycf2* gene's function remains elusive. Interestingly, its length varied across species, with *P. filipes* at 2236 amino acids, and *P. cyrtonema, P. odoratum* and *P. zanlanscianense* at 2250 and 2251 amino acids, respectively. This divergence is observed not only in other *Polygonatum* species but also in closely related plants. Such pronounced variations across several genes underscore the intricacies of *Polygonatum* species’ in the evolutionary journey. In summary, this study presents a comparative genomic exploration of complete cp genomes within *P. filipes, P. cyrtonema*, *P. zanlanscianense*, and *P. odoratum*, furnishing a foundation for species identification through molecular markers within the *Polygonatum* genus. While the species represent a fraction of the total *Polygonatum* diversity, this expanded dataset offers an enhanced grasp of cp genome attributes and variability within the *Polygonatum* genus. These insights furnish valuable evidence to enhance our comprehension of *Polygonatum* species’ phylogeny and evolution.

## Conclusion

In summary, this study encompassed the comprehensive sequencing and determination of complete cp genomes in *P. filipes*, *P. cyrtonema*, *P. zanlanscianense* and *P. odoratum*, coupled with a comparative analysis involving closely related members of the Liliaceae family. The collective findings underscored a marked conservation trend within that the *Polygonatum* genus, evident in key aspects such as the quadripartite structure, sequence length, gene count, GC content, and functional attributes of the whole cp genomes. Notably, our comparative investigation identified six regions of heightened variability, for which we meticulously designed five pairs of specific primers. The utility of the *psaJ-rpl33* and *trnS* + *trnT-psaD* barcodes emerged as promising molecular markers for species identification, evolutionary exploration, and phylogenetic analysis within *Polygonatum* species. In essence, our study contributes crucial genomic data encompassing *P. filipes*, *P. cyrtonema*, *P. zanlanscianense* and *P. odoratum*, significantly enriching our understanding of the phylogenetic landscape of *Polygonatum* species. This article bears paramount significance in the realm of genome evolution, facilitating the establishment of potent molecular markers, unraveling intricate phylogenetic relationships, and refining the classification schema within the genus *Polygonatum*.

## Methods

### Plant material and DNA extraction

Fresh leaves of *P. filipes*, *P. cyrtonema* and *P. zanlanscianense* were meticulously collected from Dazhe Town, Suichang County, Lishui City, Zhejiang Province (28°52′N, 119°11′E), while *P. odoratum* was sourced from Bozhou City, Anhui Province (33°86′N, 115°78′E). These four plant specimens were authenticated by Dr. Yuqing Ge from Zhejiang Chinese Medical University and deposited at the Medicinal Herbarium Center of Zhejiang Chinese Medical University, Hangzhou, Zhejiang Province, China (https://yxy.zcmu.edu.cn, Herbarium Code: MHCZCMU; Collector: Yuqing Ge; Email: geyuqing@zcmu.edu.cn). The specific voucher numbers for *P. filipes*, *P. cyrtonema*, *P. zanlanscianense* and *P. odoratum* are documented in Table S2. These specimens are meticulously within the Medicinal Herbarium Center of Zhejiang Chinese Medical University. For DNA extraction, total genomic DNA was procured from dried leaves that were pulverized using liquid nitrogen, following a modified cetyltrimethylammonium bromide (CTAB) protocol^70^. The quality of the final DNA was assessed using by a Nanodrop spectrophotometer (Thermo Fisher Scientific, USA), while DNA integrity was gauged via 1.0% agarose gel electrophoresis.

### Genome sequencing, assembly and annotation

Whole-genome DNA underwent sequencing using an Illumina HiSeq Platform courtesy of Genesky Biotechnologies Inc. The quality of the paired-end Illumina reads was verified using FastQC, with Fastp employed for the elimination of low-quality reads. Primarily, rigorous quality control and filtering of the original data was executed, guided by the following conditions: (i) exclusion of sequences containing more than three N bases; (ii) omission of sequences where high-quality bases (Phred score ≥ 20) accounted for less than 60%; (iii) removal of the 3' terminal low-quality base; and (iv) elimination of sequences sequence lengths below 60 bp. The filtered reads were subsequently subjected to de novo assembly utilizing the metaSPAdes algorithm, referencing the complete cp genomes of *P. cyrtonema* (NC_028429), *P. cirrhifolium* (NC_053687), *P. sp. JJ-2020* (MN906758), and *P. odoratum cultivar Dazhu* (MW248131), respectively. The GeSeq annotation tool was harnessed for comprehensive gene annotation, encompassing protein-coding genes, mRNA genes, and tRNA genes^71^. Additionally, the CPGAVAS2 software facilitated annotation of protein-coding genes^72^. Post-annotation, manual validation was performed through BLAST analysis. Circular cp genome visualizations for the four *Polygonatum* species were crafted employing the OrganellarGenome DRAW (OGDRAW) tool^73^. Ultimately, the thoroughly annotated cp genomes were duly deposited in the GenBank database, assigned Accession Numbers: MZ571521, MZ579646, MZ568930 and MZ666387.

### Comparative analysis of cp genomes

Genomic feature analysis was conducted using MEGA v11^74^, while Codon W software was employed to discern codon usage and relative synonymous codon usage (RSCU), using default parameters. RNA editing sites within protein-coding genes were identified through the RNA Editor tool, specifically targeting Plant CP genes (PREP-cp), with a cutoff value of 0.8 ^75^. For repeats analysis, SSRs in the four *Polygonatum* plants were detected using the online tool MISA. The criteria for SSR detection were set at 10 repeat units for mononucleotide repeats, 5 units for dinucleotide repeats, 4 units for trinucleotide repeats, and 3 units for tetra-, penta-, and hexanucleotide repeats ^76^. REPuter was harnessed to compute long repeats of various types (forward (F), palindromic (P), reverse (R), and complementary (C)), configured with a hamming distance of 3, a minimum repeat length of 30 bp, and a maximum computed repeat length of 50 bp ^77^. To delineate the boundaries of IR regions, the LSC/IRb/SSC/IRa boundary positions were investigated using IRscope (https://irscope.shinyapps.io/irapp/) across the cp genomes of the eight *Polygonatum* species under default settings ^78^. Subsequently, the cp genomes were aligned via MAFFT ^79^, and DnaSP v6 was employed to calculate the Ka/Ks value.

### Identification of divergence regions and PCR primers designing

Multiple sequence alignments encompassing the complete cp genomes of the eight *Polygonatum* plants were executed using MAFFT to elucidate sequence disparities. The calculation of nucleotide variability (Pi) was performed utilizing DnaSP v6, employing a window length of 600 bp and a step size of 200 bp ^80^. Distinctive primers were meticulously designed through Primer Premier 5, with a focus on mutation hotspots and adherence to conditions such as 40–60% GC content and primer lengths ranging from 15 to 30 bp.

### Phylogenetic analysis

The cp genomes were amassed from NCBI. Evaluating the cp genome divergence amongst the four *Polygonatum* plants and 16 additional plants was undertaken. Through MAFFT, the whole cp genomes were subjected to multiple alignment, followed by maximum likelihood (ML) analysis utilizing MEGA v11to construct a phylogenetic tree. The ML tree, with 1000 bootstrap replications, was generatedbased on the Kimura 2-parameter model, with *Aegilops tauschii* and *Miscanthus sinensis* serving as outgroup plants.

### Supplementary Information

Supplementary Information.

## Data Availability

The chloroplast genomes of four *Polygonatum* assembled in this study have been deposited in the National Center for Biotechnology and Information (NCBI, https://www.ncbi.nlm.nih.gov/) under the accession numbers MZ571521, MZ579646, MZ568930 and MZ666387. The other cp genomes used in this study were downloaded from the NCBI.
