# Supplementary material for: Comparative chloroplast genome analysis of four Polygonatum species insights into DNA barcoding, evolution, and phylogeny
Source: Sci Rep. 2023 Oct 1;13:16495. doi: 10.1038/s41598-023-43638-1 (PMC10543443; doi:10.1038/s41598-023-43638-1)
Supplement: Supplementary file 1 — Supplementary Information. [file 41598_2023_43638_MOESM1_ESM.pdf]

Supplementary Information

Title:

Comparative chloroplast genome analysis of four Polygonatum species insights into DNA barcoding, evolution, and phylogeny.

Table S1. The SSR types of four *Polygonatum* species

SSR type	Repeat unit	Amount			
		P.zanlanscianense	***P. filipes***	***P.cyrtonema***	***P.odoratum***
Mono	A/T	36	35	31	33
	C/G	0	1	0	1
Di	AT/TA	9	9	8	9
	GA/TC	2	2	2	2
Tri	CAG/TTA	2	2	2	2
	ATA/ATT	1	2	2	2
	CCT/TAT	0	0	0	1
Tetra	ATTG/ AATG	2	2	2	2
	GAAT/TTAA	2	2	2	2
	AATA/TTGA	2	3	2	2
	CATT/TTTA	1	1	1	1
Penta	ATAAT/ CGAAA	2	1	1	1
	TTTCG/AATTA	1	1	1	1
Hexa	ATAGTA	0	0	0	1

Table S2. The information of sample collection and DNA sequencing of four *Polygonatum* species.

**Sample**	**Location**	**Latitude (N)**	**Longitude (E)**	**Raw reads**	**Raw Bases**	**Genbank accession number**
P.filipes	Da zhe zhen, Sui chang County, Zhejiang, China	28 °52′	119 °11′	22,496,704	3,329,565,273	MZ571521
P.cyrtonema	Da zhe zhen, Sui chang County, Zhejiang, China	28 °52′	119 °11′	15,915,998	3,682,353,693	MZ579646
P.zanlanscianense	Da zhe zhen, Sui chang County, Zhejiang, China	28 °52′	119 °11′	32,147,396	4,600,981,992	MZ568930
P.odoratum	Bozhou, Anhui, China	33 °86′	115 °78′	26,691,780	3,981,228,737	MZ666387

Figure S1. The original full-length agarose gel of five pairs of primers.

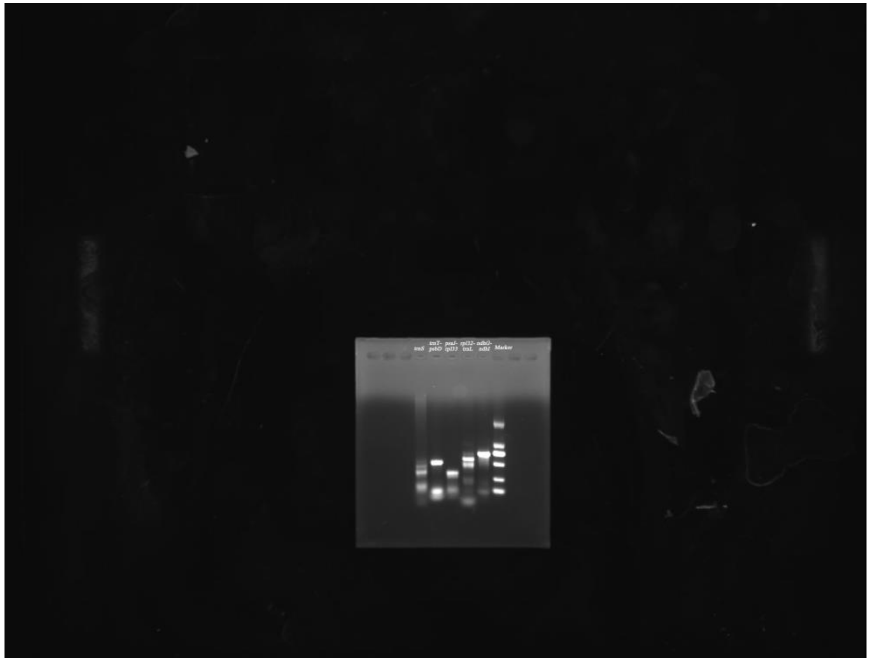

The original full-length agarose gel of five pairs of primers and from left to right were *trnS*, *trnT*-*psbD*, *psaJ*-*rpl33*, *rpl32*-*trnL* and *ndhG*-*ndhI*, respectively. *P. cyrtonema* plant was used for PCR amplification in this figure.
